# BCL9/STAT3 regulation of transcriptional enhancer networks promote DCIS progression

**DOI:** 10.1038/s41523-020-0157-z

**Published:** 2020-04-24

**Authors:** Hanan S. Elsarraj, Yan Hong, Darlene Limback, Ruonan Zhao, Jenna Berger, Stephanie C. Bishop, Aria Sabbagh, Linzi Oppenheimer, Haleigh E. Harper, Anna Tsimelzon, Shixia Huang, Susan G. Hilsenbeck, Dean P. Edwards, Joseph Fontes, Fang Fan, Rashna Madan, Ben Fangman, Ashley Ellis, Ossama Tawfik, Diane L. Persons, Timothy Fields, Andrew K. Godwin, Christy R. Hagan, Katherine Swenson-Fields, Cristian Coarfa, Jeffrey Thompson, Fariba Behbod

**Affiliations:** 1 Department of Pathology and Laboratory Medicine, The University of Kansas Medical Center, 3901 Rainbow Blvd, Kansas City, KS 66160 USA; 2 Warren Alpert Medical School of Brown University, Providence, RI 02912 USA; 3 Department of Pharmaceutical Sciences, South University, 709 Mall Blvd, Savannah, GA 31406 USA; 4 McGovern Medical School, The University of Texas Health Science Center, Houston, TX 77030 USA; 5 University of Kansas School of Medicine, The University of Kansas Medical Center, 3901 Rainbow Blvd, Kansas City, KS 66160 USA; 6 Department of Medicine, Baylor College of Medicine, One Baylor Plaza, Houston, TX 77030 USA; 7 Dan L. Duncan Cancer Center and Department of Molecular & Cellular Biology, Baylor College of Medicine, One Baylor Plaza, Houston, TX 77030 USA; 8 Lester and Sue Smith Breast Center, Baylor College of Medicine, One Baylor Plaza, Houston, TX C30 USA; 9 Department of Biochemistry and Molecular Biology, The University of Kansas Medical Center, 3901 Rainbow Blvd, Kansas City, KS 66160 USA; 10 MAWD Pathology Group, St Luke’s Health System of Kansas City, 2750 Clay Edwards Dr, Kansas City, MO 64116 USA; 11 Department of Anatomy and Cell Biology, The University of Kansas Medical Center, 3901 Rainbow Blvd, Kansas City, KS 66160 USA; 12 Department of Biostatistics, The University of Kansas Medical Center, 3901 Rainbow Blvd, Kansas City, KS 66160 USA; 13 Department of Pathology and Laboratory Medicine, MS 3045, The University of Kansas Medical Center, Kansas City, KS 66160 USA

**Keywords:** Breast cancer, Cancer prevention

## Abstract

The molecular processes by which some human ductal carcinoma in situ (DCIS) lesions advance to the more aggressive form, while others remain indolent, are largely unknown. Experiments utilizing a patient-derived (PDX) DCIS Mouse INtraDuctal (MIND) animal model combined with ChIP-exo and RNA sequencing revealed that the formation of protein complexes between B Cell Lymphoma-9 (BCL9), phosphoserine 727 STAT3 (PS-727-STAT3) and non-STAT3 transcription factors on chromatin enhancers lead to subsequent transcription of key drivers of DCIS malignancy. Downregulation of two such targets, integrin β3 and its associated metalloproteinase, MMP16, resulted in a significant inhibition of DCIS invasive progression. Finally, in vivo targeting of BCL9, using rosemary extract, resulted in significant inhibition of DCIS malignancy in both cell line and PDX DCIS MIND animal models. As such, our studies provide compelling evidence for future testing of rosemary extract as a chemopreventive agent in breast cancer.

## Introduction

It is currently believed that human Ductal carcinoma in situ (DCIS) is over diagnosed and over treated. DCIS is a non-obligate precursor to invasive breast cancer and due to recent advances in imaging technology and an increase in screening, there has been a significant increase in the rate of DCIS detection^1–4^. Despite this increase, the rate at which women present with late-stage invasive breast cancer has only marginally declined (~8%)^5^. Currently, DCIS is treated by surgery, mastectomy (for extensive disease) or lumpectomy plus radiation, and problematic anti-hormonal therapy for hormone receptor positive DCIS. Anti-hormonal therapies, while effective, are associated with many side effects, resulting in only ~50% patient compliance^6^. The indolent nature of a substantial proportion of DCIS lesions is supported by observational studies showing that untreated DCIS is followed by invasive disease in less than 50% of women^7–10^ suggesting that for many women harmful interventions may have been unnecessary. Therefore, there is a critical need for the discovery of cellular and molecular mechanisms by which some DCIS transition to invasive ductal carcinoma (IDC) which would elucidate biomarkers for DCIS risk stratification and help develop therapies for prevention of IDC.

Towards the understanding of DCIS pathobiology, we developed an animal model referred to as Mouse-INtraDuctal (MIND), which involves intraductal injection of DCIS epithelial cells, derived from patient samples or cell lines, into the mammary ducts of immunocompromised mice^11–13^. Among available models, the MIND model is the best suited to demonstrate a transition from non-invasive DCIS to IDC. Patient-derived DCIS epithelial cells when injected by the MIND method mimic patient pathology with respect to histology, biomarker expression, and progression to invasion^13^.

By utilizing the MIND model at distinct stages of transition from DCIS to IDC, we found expression of both BCL9 RNA and protein were significantly elevated at the time of progression^14^. Furthermore, we demonstrated a significant association between high nuclear BCL9 and pathological characteristics indicative of high risk DCIS and subsequent in vivo silencing of BCL9 in our DCIS MIND models led to inhibition of both DCIS invasion and reversal of epithelial mesenchymal transition (EMT)^14^. These data provide strong evidence for the role of BCL9 as a molecular driver of DCIS invasive progression which was previously unrecognized.

Even though the role of BCL9 in the Canonical Wnt cascade was previously demonstrated, there is limited research on which specific Wnt targets are regulated by BCL9 and whether BCL9 regulates other signaling pathways to drive cancer malignancy. We have demonstrated that BCL9 in a protein complex with STAT3 drives DCIS invasive progression by regulation of enhancers and enhancer associated target genes involved in cellular growth, invasion and migration. We have identified two genes linked to BCL9/STAT3 associated enhancers, integrin β3 and its associated MMP16, and have validated their role in DCIS invasive progression. Furthermore, we have demonstrated the efficacy of rosemary extract and its major ingredient carnosic acid in prevention of DCIS progression by targeting BCL9.

## Results

### BCL9 copy number and transcriptional alterations in human breast cancer

METABRIC database in cBioPortal, which includes 2,509 cases of breast cancer^15,16^, showed significantly higher BCL9 mRNA expression in luminal A, B and basal-like breast cancers compared to other subtypes (Supplementary Fig. 1a, b). Additionally, METABRIC revealed that a significantly higher proportion of basal (25.8%) and luminal A (24.9%) breast cancers exhibited *BCL9* genomic amplification (Supplementary Fig. 1c, d). In addition, analysis of The Cancer Genome Atlas (TCGA) database showed significantly lower DNA methylation in the *BCL9* promoter region (transcription start site ±3 kB) of luminal A and B breast cancers compared to control tissues (Supplementary Fig. 1e, f). Taken together, these results suggest that aberrant elevated expression of BCL9 in breast cancers is driven by *BCL9* genomic amplification and/or promoter hypomethylation. Additionally, we studied BCL9 protein expression in human DCIS tissue microarrays (TMAs) consisting of 60 DCIS with associated IDC (DCIS-IDC) and 30 pure DCIS cases. Immunofluorescence (IF) staining of TMAs was performed using BCL9-specific antibodies and nuclear intensity was measured by the Metamorph^®^ software. Nuclear BCL9 was significantly higher in both the IDC and DCIS regions of DCIS-IDC samples compared to either pure DCIS or adjacent normal tissue (Supplementary Fig. 1g). In summary, increased expression of BCL9, as observed in a significant fraction of breast cancer patients, may predict DCIS with invasive potential. Subsequently, BCL9 protein expression by Western blot was investigated in five breast cancer cell lines including: MCF7 (ER+ PR+), T47D (ER+ PR+), CCH1 (DCIS Basal), DCIS.COM (DCIS Basal), SUM225 (DCIS HER2 + ) as well as MCF10A (immortalized, non-tumorigenic mammary epithelial cell line), and 293 T (kidney embryonic cell line). The data showed highest BCL9 expression in MCF7 and DCIS.COM but moderate expression in SUM225 compared to MCF10A, 293 T, CCH1 or T47D (Supplementary Fig. 2a, b). Furthermore, fluorescence in situ hybridization (FISH) showed *BCL9* amplification in DCIS.COM and SUM225 (Supplementary Fig. 2b). We chose to study DCIS.COM and SUM225 for our subsequent studies as the cell lines represent two distinct subtypes of DCIS with respectively high to moderate level expression of BCL9.

### BCL9 regulation of both STAT3 direct targets and upstream regulators

In order to explore a mechanism by which BCL9 may regulate malignant transition of human DCIS, Reverse Phase Protein Analysis (RPPA) was performed. RPPA uses 200+ validated antibodies to detect differential expression of proteins relevant to cancer. We compared RPPA results in DCIS.COM and SUM225 cell lines, which expressed knockdown of BCL9 (BCL9-KD) and non-silencing (NS) controls (Supplementary Fig. 2c). RPPA analysis revealed that BCL9 KD resulted in downregulation of a number of oncoproteins including p-AKT, p-EGFR, p-p70S6K, integrin β3, p-Src, p-STAT3, and p-mTOR (Supplementary Fig. 3a, b). Interestingly, Ingenuity pathway analysis (IPA)^17^ revealed that a number of these proteins were either direct STAT3 targets, i.e., integrin β3, Cox-2, FoxO1, p-c-Jun, or served as upstream regulators of STAT3 including EGFR, IGF, PDGF, HER2, ERK/MAPK, HGF, ILK, IL-6, and JAK/STAT pathways (Supplementary Fig. 3a, b, Supplementary Data 1). BCL9 downregulation was also associated with upregulation of tumor suppressors such as BAD, CDKN1B, and PTEN (Supplementary Fig. 3a, b, Supplementary Data 1). These results supported the notion that BCL9 was involved in regulating the expression of a number of oncoproteins, some of which were either direct STAT3 transcriptional targets or served as upstream regulators of STAT3 pathway.

### BCL9 interaction with phosphoserine 727 STAT3 (PS-727-STAT3)

**Fig. 1:**
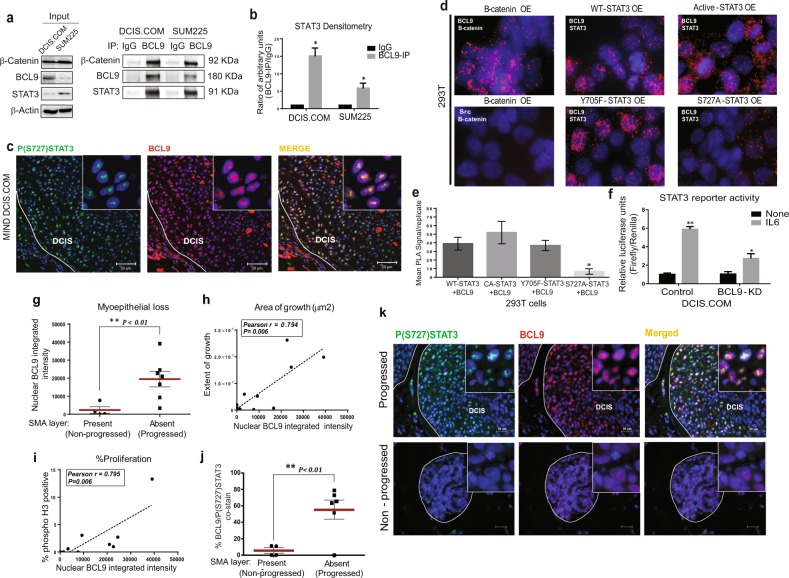
BCL9/STAT3 nuclear co-expression in DCIS MIND xenografts was associated with DCIS invasive progression. **a** Whole-cell extracts of DCIS.COM and SUM225 cells were immunoprecipitated using an anti-BCL9 antibody, followed by western blot analysis using anti-β-catenin, anti-BCL9, anti-STAT3 and control IgG antibodies. **b** Densitometry analysis of STAT3 protein in BCL9 pull-down normalized to IgG control in DCIS.COM and SUM225 cells (*n* = 3, *P < 0.05). **c** Representative IF images of DCIS.COM MIND xenografts 8 weeks post-intraductal injection. Staining represents P(S727) STAT3 (green), BCL9 (red), and Hoechst (blue). Areas of colocalization of BCL9 and P(S727) STAT3 in the merged image shown in yellow. **d** PLA using Duolink® PLA kit in 293 T cells transfected with BCL9 overexpression (OE) and either β-catenin, or wild-type (WT)-STAT3, constitutively active (CA)-STAT3, mutant Y705F-STAT3, mutant S727A-STAT3. The antibodies used are indicated in each panel. Anti-β-catenin and anti-BCL9 antibodies were used as PLA-positive controls. Src and β-catenin antibodies were used for PLA-negative control. PLA signals are detected by fluorescence microscopy and appear as red discrete spots. **e** Bar graphs represent quantification of PLA signals in three biological replicates. PLA signals were analyzed using NIS Elements Analysis Software. **f** STAT3 reporter assay in BCL9-knockdown (KD) DCIS.COM with and without IL6 stimulation. Data were analyzed using unpaired *t*-test multi-group comparison (asterisk represents a statistically significant difference; *P* < 0.05; *n* = 3 replicates per group). **g** Whisker plots represent distribution of nuclear BCL9 expression in Patient-derived (PDX) DCIS MIND xenografts that showed invasive progression (*n* = 7) vs. those that remained non-invasive (*n* = 4). **h**, **i** Pearson correlation between nuclear BCL9 expression and extent of growth (**h**) or proliferation rate (**i**) in PDX DCIS MIND xenografts (*n* = 9). **j** Whisker plots representing the distribution of nuclear BCL9-P (S727) STAT3 co-expression in progressed (*n* = 6) vs. non-progressed (*n* = 4) PDX DCIS MIND xenografts (asterisk represents statistically significant difference in mean values; *P* < 0.05). **k** Representative IF images of nuclear BCL9 (red) and P(S727) STAT3 (green) expression and their expression (yellow) in progressed vs. non-progressed primary DCIS MIND xenografts. Data are presented as mean ± SEM.

To examine a protein interaction between BCL9 and STAT3, whole-cell extracts of DCIS.COM and SUM225 were co-immunoprecipitated (Co-IP) with anti-BCL9 and anti-STAT3 antibodies followed by Western blot using anti-STAT3, anti-BCL9 and anti-P(Y705) STAT3 antibodies. As shown in Fig. 1a, b, BCL9 and STAT3 showed Co-IP in both cell lines. A reverse IP using STAT3 pull-down also confirmed that STAT3 and BCL9 were part of the same protein complex (Supplementary Fig. 4a). To confirm STAT3-BCL9 association in vivo, IF staining was performed on DCIS.COM and SUM225 MIND xenografts in which DCIS epithelial cells were injected intraductally into immunocompromised mice and studied as they progressed to IDC. We previously reported that DCIS.COM MIND xenografts progressed from DCIS to invasive lesions in 8–10 weeks post-intraductal injection^12^. At this time point, IF staining with anti-PS-727-STAT3 and anti-BCL9 antibodies revealed cellular colocalization of STAT3 and BCL9 in the nuclei of DCIS.COM (Fig. 1c) and SUM225 xenografts (data not shown).

BCL9 and STAT3 associations were further confirmed by Proximity Ligation Assay (PLA) using Duolink^R^. PLA using Duolink^®^ PLA kit was performed on DCIS.COM cells using anti-β-catenin and anti-BCL9 antibodies (Supplementary Fig. 4b**:** top), or anti-PS-727-STAT3 and anti-BCL9 antibodies (Supplementary Fig. 4b**:** bottom). The resulting red spots confirmed a close association between BCL9 and PS-727-STAT3 in DCIS.COM cells and suggested that these proteins may be direct binding partners. PLA was also used in 293 T cells transfected with BCL9 (OE) and either β-catenin, non-mutated wild-type (WT)-STAT3, constitutively active (CA)-STAT3, phosphorylation mutants Y705F-STAT3 or S727A-STAT3. Anti-β-catenin and anti-BCL9 antibodies were used as PLA-positive controls. Src and β-catenin antibodies were used as PLA-negative control (Fig. 1d). PLA signals were then quantified and reported as bar graphs (Fig. 1e). These experiments demonstrated a binding interaction between BCL9 and STAT3. Furthermore, the data showed that the binding interaction between BCL9 and STAT3 may be mediated by PS-727-STAT3 as there was a significant reduction in PLA signals when PS-727-STAT3 was mutated, while there was no change in BCL9/STAT3 interactions when Y705-STAT3 was mutated.

To examine a potential role of BCL9 in STAT3 transcriptional activity, an inducible STAT3-responsive firefly luciferase construct was utilized. As shown in Supplementary Fig. 4c, BCL9 plus CA-STAT3 significantly increased STAT3 transcriptional activity compared to CA-STAT3 or BCL9 alone (*P* < 0.01). Furthermore, the same STAT3 luciferase construct was electroporated into DCIS.COM and SUM225 cells; both control and BCL9 KD cells were then treated with and without IL6 to activate the endogenous STAT3. When treated with IL6, there was a significant reduction in STAT3 reporter activity in BCL9-KD DCIS.COM cells compared to control cells (Fig. 1f; *P* < 0.05). These results collectively suggest that BCL9 interactions with STAT3 may either directly or indirectly induce STAT3 transcriptional activity.

### BCL9/PS-727-STAT3 nuclear co-expression may predict DCIS with invasive potential

By utilizing patient-derived (PDX) DCIS MIND models, our studies demonstrated that BCL9/PS-727-STAT3 nuclear colocalization correlated with DCIS invasive progression. The PDX DCIS MIND xenografts mimic patient pathology with respect to biomarker expression and histology, and a subset (13/24 patient cases = 50%) develop invasive lesions 6–12 months following transplantation (Supplementary Fig. 4d and data not shown). We performed IF staining on a subset of PDX DCIS MIND xenografts to assess nuclear BCL9 expression in those PDX DCIS MIND models that exhibited invasive progression compared to those that remained non-invasive. PDX DCIS MIND xenografts that showed invasive progression by the loss of SMA, expressed significantly higher BCL9 nuclear expression (Fig. 1g), showed significantly higher area of growth (Fig. 1h) and exhibited higher proliferation rate (Fig. 1i). As shown in Fig. 1j-k, PDX DCIS MIND models that showed invasive progression exhibited significantly higher nuclear BCL9/PS-727-STAT3 co-expression (Fig. 1j, k) compared to those that remained non-invasive. Among the non-progressed group, two out of five cases, were ER+/PR+ and among the progressed group, four out of seven cases, were ER+/PR+ (Supplementary Table 1). Supplementary Fig. 4e shows loss of SMA staining surrounding a representative progressed PDX DCIS MIND xenograft (high BCL9/PS-727-STAT3 co-expressing lesion) compared to a representative non-progressed PDX DCIS MIND xenograft (low BCL9/PS-727-STAT3 co-expressing) that showed an intact SMA layer. These data support that BCL9/PS-727-STAT3 nuclear co-expression may serve as a biomarker of DCIS with invasive potential. Furthermore, BCL9/PS-727-STAT3 nuclear co-expression and biomarker potential may be independent of DCIS hormone receptor status.

### BCL9 and PS-727-STAT3 form protein complexes on chromatin enhancer sequences to regulate human DCIS invasive progression

**Fig. 2:**
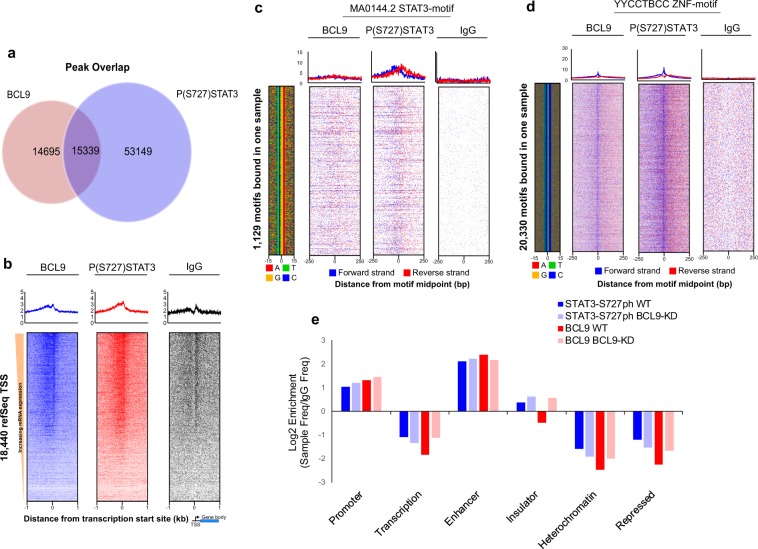
BCL9 and PS-727-STAT3 protein complexes associate with non-STAT3 transcription factors and show co-occupancy with a higher frequency on chromatin enhancers. **a** Venn diagram demonstrating the number of co-occurring BCL9 and PS-727-STAT3 peaks. **b** Enrichment of BCL9 and PS-727-STAT3 co-occurring peaks on promoters of refSeq genes. **c**, **d** Motif analysis of BCL9 and PS-727-STAT3 co-occurring peaks. Analysis revealed presence of weak co-occurring peaks at STAT3 motifs (**c**), while there was a significant number of co-occurring peaks at YYCCTBCC representative of zinc-finger binding motifs (**d**). **e** Chromatin state enrichment using ChromHMM inferred states in HMEC cells. Log2-fold frequency of co-occurring peaks for BCL9 and PS-727-STAT3 compared to control (IgG) peak frequencies.

In order to determine if BCL9 and PS-727-STAT3 complexes associate with chromatin, Chromatin immunoprecipitation (ChIP)-exo experiments were performed using anti-BCL9 and anti-PS-727-STAT3 antibodies. ChIP-exo is a variation of ChIP-sequencing designed to improve sensitivity and positional resolution. It uses lambda exonuclease to digest sonicated chromatin to the nearest base pair resolution of protein-DNA crosslinking points^18^. By doing so, structural insights into protein complex organizations are obtained. We also simultaneously performed RNA sequencing of DCIS.COM non-silencing (NS) control and BCL9 KD cells. RNA sequencing was performed in order to simultaneously assess the role of DNA regulatory elements (i.e., chromatin regions that showed common peaks of BCL9 and PS-727-STAT3) and BCL9 regulation of gene expression. ChExMix was utilized to analyze the ChIP-exo data. ChExMix identifies protein-DNA binding events by combining DNA sequences and unique distribution of sequence tags around known transcription factor binding motifs^19^. In order to evaluate PS-727-STAT3 and BCL9 common peaks on chromatin, sample peaks were expanded to a 100 base pair window centered on the midpoint. This comparison showed that ~22% of the PS-727-STAT3 and BCL9 peaks were overlapping (15,339 common peaks) (Fig. 2a). In order to evaluate a possible BCL9/PS-727-STAT3 protein interactions on chromatin, median peak-peak distances were evaluated. This analysis showed a low peak-peak median distance of only 94 base pairs between PS-727-STAT3 and BCL9 on the overlapping regions of chromatin, suggesting a potential binding interaction between PS-727-STAT3 and BCL9 (data not shown). Additionally, RNA-seq data for MCF7 generated by the ENCODE project was used to sort RefSeq transcription start sites (TSS) by steady-state expression levels. The aligned sequence reads were piled up relative to TSS. A strong BCL9/PS-727-STAT3 co-occurring peaks were observed on the promoters of RefSeq targets (Fig. 2b). In order to better identify putative protein–protein interactions between STAT3, BCL9, and other known transcription factors, peaks were intersected with the genomic location of known transcription factor binding motifs^20^. Using the published JASPAR motifs^20,21^, the FIMO software was used to identify all motif occurrences in the hg19 reference genome. Motifs were then filtered to overlap within 100 bp of overlapping peaks from the ChIP-exo samples. As expected, PS-727-STAT3 peaks showed strong enrichment at STAT3 motifs. However, BCL9 did not appear to be co-occurring with STAT3 peaks (Fig. 2c). Interestingly, our analysis found that PS-727-STAT3 and BCL9 overlapping peaks were enriched on the predicted binding motifs of other transcription factors including ETS (ELK4 and ELF5), C/EBP family members, AP-1, REL and a large number of zinc-finger containing (YYCCTBCC) transcription factors (~20,000 binding motifs) (Supplementary Fig. 5a–e and Fig. 2d). These data demonstrate that BCL9/PS-727-STAT3 complexes associate with chromatin through binding motifs of non-STAT3 transcription factors.

### BCL9 and PS-727-STAT3 co-occurring peaks on enhancers support their role as active regulatory proteins

**Fig. 3:**
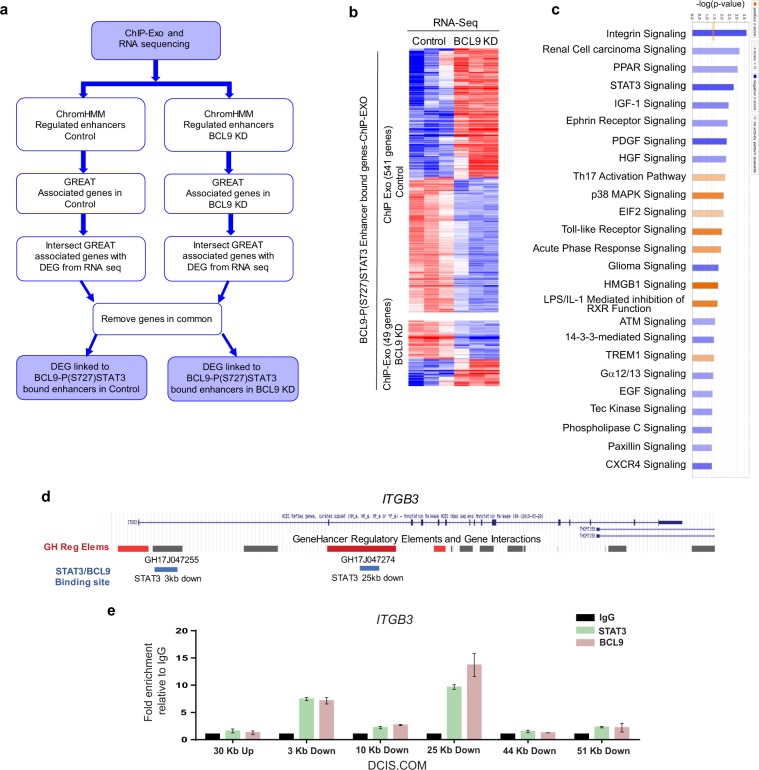
Analysis of BCL9/P(S727)STAT3 regulated targets involved in DCIS invasive progression by integrating ChIP-exo and RNA-seq data. **a** Schematic of ChIP-exo/RNA sequencing analysis in DCIS.COM cells. ChIP-exo was performed to identify BCL9/PS-727-STAT3 co-enriched sequences of chromatin. ChromHMM inferred epigenomic states in HMEC cells were used to annotate the co-occurring sequences (enhancers vs. promoters, etc). Enhancer sequences were analyzed by GREAT to find enhancer associated genes and gene ontologies. Enhancer associated genes were then analyzed against RNA sequencing data, comparing BCL9 control to KD cells. **b** Analysis of BCL9/PS-727-STAT3 co-occurring peaks and their associated genes against differentially expressed genes in our RNA-seq data comparing DCIS.COM control and BCL9 KD cells. This analysis showed 541 differentially regulated genes in the control cells compared to only 49 genes in the BCL9 KD cells. **c** Ingenuity pathway analysis (IPA) of the overlapped genes showed BCL9 regulation of a number of signaling pathways involved in cancer progression and cellular migration and invasion including integrin signaling, STAT3 and growth factor signaling (HGF, PDGF, and IGF). For the IPA analysis a Z-score cutoff of 1 was used (−log *P*-value higher than 1.3 is considered significant). **d** Human *ITGB3* coding region and its associated enhancers (grey bars) and promoters/enhancers (red bars) regions from GeneHancer. The blue bars demonstrate sites of BCL9 and STAT3 co-occurring peaks confirmed by ChIP-qPCR. **e** Anti-STAT3 and anti-BCL9 ChIP followed by qPCR for two enhancer regions near the *ITGB3* gene. Enrichment for STAT3 and BCL9 binding is reported as fold-change relative to IgG. Graph represents one of five independent experiments. Data are presented as mean ± SEM of the technical replicate of the representative experiment.

Chromatin state enrichment prediction was performed using the ChromHMM software and previously reported by the Encode consortium. ChromHMM is a software program for annotating chromatin states (i.e., active enhancers vs. promoters) using a multivariate hidden Markov model that explicitly models the combination of marks (i.e., histone modifications). Chromatin states are defined based on different combination of histone modifications corresponding to different functional regions. Chromatin states inferred using ChromHMM in human mammary epithelial cells (HMEC) were used to annotate chromatin sequences that showed PS-727-STAT3 and BCL9 co-occurring peaks^22,23^. ChEx-Mix peaks were called on the IgG-negative control in order to determine a background in this cell line. The log2-fold-change of peak frequencies for each sample divided by control peak frequencies is shown in Fig. 2e. The frequency of co-occurring PS-727-STAT3 and BCL9 peaks were significantly higher on enhancers compared to IgG-negative control (>2 log2-fold). Furthermore, the frequency of co-occurring PS-727-STAT3 and BCL9 peaks were >1 log2-fold higher on enhancers compared to promoters. However, the PS-727-STAT3 and BCL9 co-occurring peaks were de-enriched at the predicted heterochromatin and repressed regions. These data support that BCL9 and PS-727-STAT3 protein complexes may serve as enhancer active regulatory proteins. In order to link chromatin enhancer sequences bound by BCL9 and PS-727-STAT3 to their associated target genes, Genomic Region Enrichment of Annotations Tool (GREAT) was used. GREAT is a new generation tool aimed at annotating cis-regulatory regions of chromatin and the genes they regulate^24^. GREAT assigns each gene a regulatory domain consisting of a basal domain that extends 5 kb upstream and 1 kb downstream from its transcription start site and an extension up to 1MB. Moreover, GREAT incorporates results from three-dimensional conformation capture studies, radiation hybrid maps and other emerging approaches for annotation of cis-regulatory genomic sequences. Enhancer sequences that showed co-occurring peaks of BCL9/PS-727-STAT3 were uploaded to the GREAT webserver in order to find their associated genes and Gene Ontologies (GO) (http://great.stanford.edu/public/html/). We also analyzed the results against the differentially expressed genes in our RNA sequencing experiments (comparing DCIS.COM cells BCL9 NS control vs. BCL9-KD) (Fig. 3a). Interestingly, analysis of the enhancer regulated genes against our RNA sequencing dataset showed 541 differentially expressed genes in the control cells compared to only 49 genes in the BCL9 KD cells (Fig. 3b, Supplementary data 2). IPA analysis of the differentially expressed genes showed significant downregulation of a number of pathways involved in cancer progression, including cellular migration and invasion, integrin signaling (integrin β3), STAT3 and growth factor signaling (HGF, PDGF, and IGF) (Fig. 3c). Based on these results we propose that BCL9 KD resulted downregulation in transcription of a number of enhancer associated target genes involved in DCIS malignancy (Supplementary data 2).

### BCL9 regulation of two of its downstream targets, integrin β3 and MMP16, regulate DCIS invasive progression

As described previously, RPPA and RNA sequencing studies showed integrin signaling as a top significantly downregulated pathway in BCL9-KD cells compared to NS control (Supplementary Fig. 3 and Fig. 3). Subsequent ChIP-exo and RNA sequencing studies also showed integrin β3 to be linked to BCL9/PS-727-STAT3 co-enriched enhancer sequences (Fig. 3). To validate these results, we performed flow cytometry analysis of known surface molecules implicated in cancer progression including frizzled 7 (FZD7), CD44, MUC-1, and integrins α2, β4, αvβ3, β3, and α4. As shown, among the surface markers tested by flow cytometry, DCIS.COM cells showed a significant reduction in surface expression of integrin αVβ3, β3, and α4 with BCL9-KD (*P* < *0.05*) (Supplementary Fig. 6a). To determine whether BCL9 regulated the expression of integrin β3 through canonical Wnt signaling, we stimulated DCIS.COM BCL9 NS and KD cells with Wnt3 ligand and measured integrin β3 expression by QPCR. As shown, Wnt3A did not increase the expression level of integrin β3, while BCL9-KD resulted in a significant reduction in both baseline and Wnt3A stimulated expression (Supplementary Fig. 6b, c). In SUM225, surface expression of integrin αVβ3 did not change with BCL9-KD (Supplementary Fig. 6d). Additionally, BCL9 did not regulate the expression of integrin αV, the heterodimer binding partner of integrin β3, in DCIS.COM or SUM225 cells (Supplementary Fig. 6e, f). Previously published ChIP-seq experiments showed several STAT3 binding motifs in the cis-regulatory regions of *ITGB3*^25^. We performed ChIP-qPCR experiments and confirmed the binding of both BCL9 and STAT3 on two of the six STAT3 binding motifs, 3 kb and 25 kb downstream of *ITGB3* coding regions (Fig. 3d, e). The same two regions have been designated by GeneHancer as *ITGB3* enhancer elements with a high confidence scores (Fig. 3d: Blue bars)^26^. These data further confirm that BCL9/PS-727-STAT3 co-occurring peaks on enhancers regulate the transcription of target genes such as integrin β3 previously implicated in cancer cell migration and invasion.

**Fig. 4:**
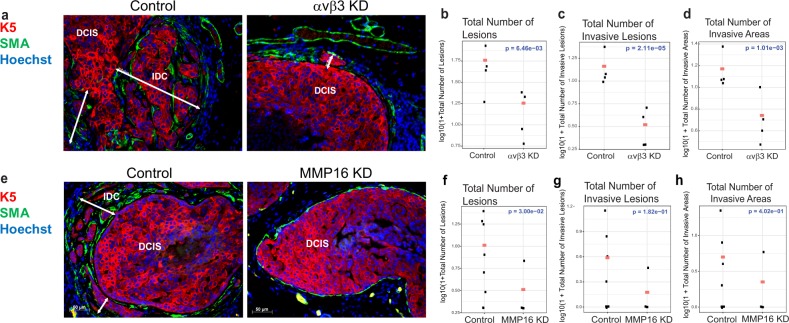
BCL9 regulation of two of its downstream targets, integrin β3 and MMP16, drive DCIS invasive progression. **a**, **e** Representative IF images of DCIS MIND xenografts generated by intraductal injection of DCIS.COM control and αVβ3 KD (**a**) or MMP16 KD (**e**). DCIS lesions are stained with human specific keratin 5 (K5) (red), SMA (green), and Hoechst (blue). **b**–**d** Plots of log-transformed number of lesions (**b**), invasive lesions (**c**), and invasive areas (**d**), in control and αvβ3 KD xenografts analyzed using a negative binomial model. The red dots indicate the response predicted by our models for each group (*n* = 4–6). Integrin αvβ3 KD resulted in a significant reduction in DCIS invasion by all three parameters (*P* < 0.05). **f**–**h** Plots of log-transformed number of lesions (**f**), invasive lesions (**g**), and invasive areas (**h**), in control and MMP16KD analyzed using a negative binomial model. The red dots indicate the response predicted by our models for each group (*n* = 4–9). MMP16 KD resulted in a significant reduction in the number of DCIS lesions only (*P* < 0.05).

To functionally validate the role of integrin β3 in DCIS invasion, MIND xenografts were generated using αVβ3-KD and NS control DCIS.COM cells followed by mammary gland analysis 8 weeks post-intraductal transplantation. As demonstrated by the lack of αVβ3 staining shown in Supplementary Fig. 6g, successful in vivo KD was achieved. A significant association was found between αVβ3 knockdown and a reduction in total number of lesions, the number of invasive lesions, and the number of invasive areas (Fig. 4a–d). However, integrin αVβ3-KD had no effect on cellular proliferation measured by phosho-histone-H3 (data not shown).

Previous studies demonstrated that integrin αVβ3 association with matrix metalloproteinases, MMP2^27^ and MMP9^28^, resulted in MMP activation leading to cellular invasion. Thus, we investigated whether BCL9 also regulated the expression and/or activity of any MMPs. The combined MMP activity of MMPs 1, 2, 3, 7, 8, 9, 12, 13, 14, and 16 was measured in both DCIS.COM and SUM225 cells with and without BCL9 KD (Supplementary Fig. 7a). Interestingly, BCL9-KD significantly reduced MMP activity in DCIS.COM (*P* < *0.05*), but not in SUM225 cells. To determine the role of BCL9 on specific MMPs, either direct STAT3 and/or canonical Wnt targets, qPCR was performed on MMP2, 3, 7, 9, 11, and 16. Among the MMPs tested, BCL9-KD significantly decreased baseline MMP16 mRNA levels in DCIS.COM cells as well as BCL9-KD cells treated with 500 ng/ml Wnt3A (Supplementary Fig. 7b). In SUM225 cells, BCL9-KD showed no effect on *MMP16* mRNA levels compared to control cells, even when stimulated with Wnt3 and a DKK-inhibitor (WAY262611) (data not shown). Western blot analysis of cell lysates of BCL9-KD and control DCIS.COM cells for MMP16 protein expression also showed a reduction in MMP16 levels in BCL9-KD compared to control cells (Supplementary Fig. 7c). To determine the role of MMP16 on invasion, lentiviral mediated knockdown of MMP16 was performed in DCIS.COM cells only, since SUM225 cells did not show any change in *MMP16* mRNA levels with BCL9-KD (Supplementary Fig. 7a). As shown in Supplementary Fig. 7d, MMP16-KD using 3 different shRNAs resulted in a significant reduction in MMP16 protein levels in DCIS.COM cells and showed consistent inhibition of invasion in vitro (data not shown). We established MIND xenografts by injecting MMP16-shRNA1 KD and control DCIS.COM cells intraductally. We collected the glands at 8 weeks and performed IF staining using MMP16 and K5 antibodies to confirm knockdown (Supplementary Fig. 7e). IF staining for human K5 and SMA was performed to assess invasion. A significant association was found between MMP16-KD and the total number of lesions (Fig. 4e; *P* = 3.00e-02). Although not statistically significant, we also found a large reduction in the number of invasive lesions and the number of invasive areas associated with MMP16-KD (Fig. 4g, h). Furthermore, MMP16-KD resulted in a significant reduction in the rate of proliferation in DCIS.COM as assessed by IF for phospho-H3 (data not shown). These data support that BCL9 regulation of integrin β3 and MMP16 played a key role in DCIS invasive progression.

**Fig. 5:**
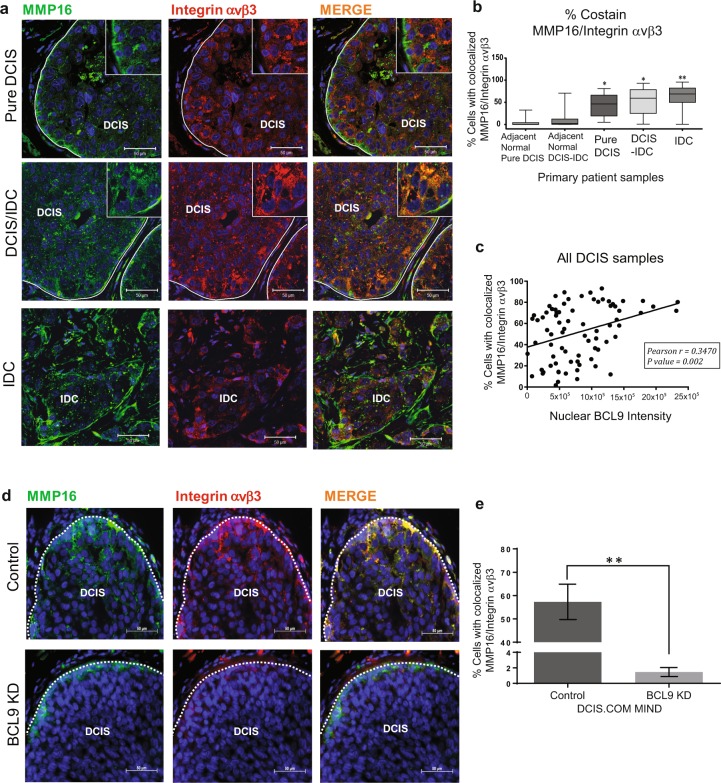
DCIS malignant progression correlates with Integrin αvβ3-MMP16 co-expression and nuclear BCL9 in DCIS. **a** Representative IF images of primary patients TMA sections stained with MMP16 (green), integrin αVβ3 (red), and Hoechst (blue) show colocalization of MMP16 and integrin αVβ3 (yellow areas) in DCIS and DCIS/IDC lesions. Scale bars = 50 μm. **b** Representative box plots of % αVβ3-MMP16 co-staining on TMA tissues. The box plots represent means and the bars represent standard error of the mean. The groups include DCIS in pure DCIS cases, DCIS component and IDC component in DCIS/IDC cases as well as normal adjacent tissues. **c** Scatter plot analyzing a correlation between αVβ3-MMP16 co-expression and nuclear BCL9 in DCIS lesions (*n* = 78). Pearson correlation = 0.3470, *P* < 0.05. **d** Representative IF images of DCIS.COM Control and BCL9 KD MIND xenografts stained with anti-MMP16 (green), anti-integrin αVβ3 (red), and Hoechst (blue) show colocalization of MMP16 and integrin αVβ3 (yellow areas) in the DCIS lesions in control (top row) and loss of expression as well as colocalization in BCL9 KD (bottom row). Scale bars = 50 μm. **e** Bar graph represents % αVβ3-MMP16 co-staining in control and BCL9 KD MIND xenografts. Data are presented as mean ± SEM. Data were analyzed using unpaired two-tailed *t*-test comparison (double asterisks represent statistically significant difference; *P* < 0.01; *n* = 3 replicates per group).

To investigate BCL9 co-regulation of integrin αVβ3 and MMP16 in vivo, IF staining was performed on tissues from 60 DCIS with associated IDC (DCIS-IDC) and 30 pure DCIS using the same TMA set described in Supplementary Fig. 1g. These studies showed colocalization of these proteins in pure DCIS lesions and in DCIS-IDC lesions (Fig. 5a). However, the percentage of cells that showed colocalization of MMP16 and integrin αVβ3 was significantly higher in IDC lesions compared to DCIS lesions (Fig. 5b and Supplementary Fig. 7f). A weak binding interaction was also found between MMP16 and integrin αVβ3 in vitro by IP experiments (data not shown). Next, to test whether nuclear BCL9 expression was associated with colocalization of MMP16/integrin αVβ3, Pearson correlation analysis was performed on the DCIS tissue samples. As shown in Fig. 5c, a significant positive correlation was found between nuclear BCL9 expression and cellular MMP16 and integrin αVβ3 colocalization. Furthermore, as shown in Fig. 5d, e, there was a significant loss of integrin αVβ3 and MMP16 expression and colocalization in BCL9 KD compared to control DCIS xenografts. Based on these data, we propose that BCL9 co-regulation of two of its downstream targets, MMP16 and integrin αVβ3, play a key role in DCIS invasive progression.

### Therapeutic targeting of BCL9 by rosemary extract (RE) and its major ingredient, carnosic acid (CA), inhibited DCIS invasive progression

**Fig. 6:**
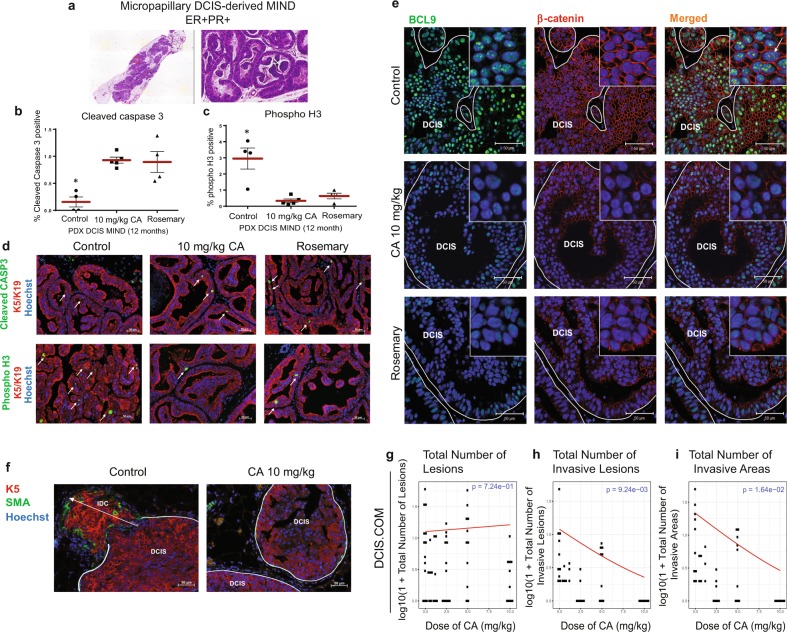
Administration of Rosemary extract and its major ingredient, carnosic acid (CA), to DCIS MIND animals resulted in a significant inhibition in DCIS progression. DCIS MIND xenografts were generated by intraductal injection of patient DCIS epithelial cells obtained from surgical and/or biopsy samples. Twelve months post-transplantation, DCIS MIND xenografts were assigned to three groups: Pretreatment control (*n* = 3), CA-treated at 10 mg/kg/day (*n* = 3), and rosemary extract treated (*n* = 3) (20% CA: equivalent to CA at 10 mg/kg/day). Treatments were administered daily through oral gavage and continued for 14 days. **a** Scanned image of H&E on a section from a PDX DCIS MIND xenograft (left) and a ×20 zoomed image (right) demonstrating the formation of micropapillary DCIS lesions in xenografts resembling the original patient’s histopathology. **b, c** Whisker plots depicting the distribution of cells expressing cleaved caspase 3 (**b**) and phospho-H3 (**c**) in CA, and rosemary extract treated DCIS xenografts compared to pretreatment controls. Data were analyzed using unpaired *t*-test with multi-group comparison (*represent statistically significant difference; *P* < 0.05; *n* = 4–5 replicates per group). **d** Representative IF staining of cleaved caspase 3 (CASP3) (top; green), phospho-H3 (bottom; green), human specific keratins (K5 and 19) (red) and Hoechst (blue) in control, CA and rosemary treated PDX DCIS MIND xenografts. White arrows point to positive cells. **e** IF staining of β-catenin (red), BCL9 (green), and Hoechst in control, CA and rosemary treated PDX DCIS MIND xenografts showing decreased intensity of nuclear β-catenin and BCL9 in the DCIS cells from CA and rosemary treated xenografts. **f** Representative IF images of CA-treated DCIS.COM MIND xenografts, with K5 (red), smooth muscle actin (SMA) (green), and Hoechst. **g**–**i** Plots of log-transformed number of lesions (**g**), invasive lesions (**h**), and invasive areas (**i**), in DCIS.COM MIND xenografts treated with CA or vehicle control. The red lines indicate the response predicted by our models for each dosage (*n* = 4–9, *P* < 0.05 for **h** and **i**). Data are presented as mean ± SEM.

We then examined whether pharmacologic inhibition of BCL9 may prevent DCIS to IDC transition. A study by de la Roche et al.^29^ screened for small molecular inhibitors of β-catenin binding to BCL9. Their screen found CA, a natural compound in RE, induced proteasomal degradation of active β-catenin and attenuated BCL9/β-catenin dependent transcription. We tested the efficacy of CA and RE in prevention of invasive progression in our PDX and cell line DCIS animal models. Three patient cases were selected for our studies and were classified based on BCL9 expression status with IF staining into BCL9-high (Supplementary Table 1, Case #12), and BCL9-low (Supplementary Table 1**:** Case #2 and #3). Primary DCIS cells were injected intraductally, and the xenografted mice were followed for 12 months during which the epithelial cells formed in situ lesions inside the mouse mammary ducts and acquired the pathologic features resembling the original patient DCIS lesions. Figure 6a shows a representative hematoxylin and eosin (H&E) staining for Case #12 MIND xenograft (Intermediate grade micropapillary DCIS, ER 100% PR 40%). Two weeks before sacrifice, the animals were assigned into three groups: pretreatment control, CA-treated (10 mg/kg), and RE treated (20% CA). Treatments were administered daily by oral gavage and continued for 14 days. To assess for DCIS progression in PDX DCIS models, proliferation marker phospho-H3, and apoptosis marker, cleaved caspase 3 were analyzed. As shown, CA and RE treated mice showed a significant increase in cleaved caspase 3 (Fig. 6b, d) and a significant decrease in proliferation (phospho-H3) (Fig. 6c, d) in the BCL9-High PDX MIND (Case 12), compared to pretreatment control. While, BCL9-low expressing PDX DCIS MIND (Cases 2 and 3) showed no difference in proliferation or apoptosis with treatment (data not shown). Finally, PDX MIND xenografts were examined for BCL9 and β-catenin expression. As shown in Fig. 6e, there was a significant loss of nuclear BCL9 and β-catenin expression in DCIS cells exposed to CA and RE in PDX DCIS MIND lesions.

To test the efficacy of CA on prevention of DCIS invasive progression, DCIS.COM and SUM225 MIND xenografts were administered the agent by oral gavage. After 14 days of treatment, mammary glands were harvested and assessed for invasion using IF staining (Fig. 6f). There was a dose dependent reduction in the number of invasive lesions (*P* = 9.24e-03), and the number of invasive areas (*P* = 1.64e-02) (Fig. 6h–i), while a statistically significant reduction was not found in the total number of lesions (*P* = 7.24e-01) (Fig. 6g). However, SUM225 MIND xenografts showed a trend to decrease in invasion with CA (data not shown), which might be explained with the higher IC50 observed in our in vitro studies (data not shown). In addition, the liver, kidneys and heart of CA-treated mice were examined by a pathologist and showed no evidence of tissue damage at the highest administered dose (Supplementary Fig. 8a).

DCIS.COM MIND xenografts were also examined for the effect of CA on BCL9 and β-catenin expression in vivo (Supplementary Fig. 8b). IF staining using BCL9 and β-catenin specific antibodies showed a significant reduction in BCL9 and β-catenin protein expression. A recent study demonstrated that CA binds the ligand-binding domain of androgen receptor (AR) and degrades AR via endoplasmic reticulum (ER) stress-mediated proteasomal degradative pathway^30^. We speculate that the same mechanism may also promote the degradation of β-catenin and BCL9 in DCIS cells, although further studies are needed to confirm this mechanism in our models. Additionally, we tested the effect of CA administration on the expression and colocalization of integrin αVβ3 and MMP16. As shown in Supplementary Fig. 8c–d, administration of CA was associated with a significant loss in the expression and colocalization of integrin αVβ3 and MMP16. These data support that CA/RE administration resulted in loss of BCL9 expression and subsequent downregulation of BCL9 targets, integrin αVβ3 and MMP16, two key mediators of DCIS invasive progression.

## Discussion

Previous studies supported the oncogenic role of BCL9 in cancers by serving as a co-activator of β-catenin^31–33^. However, emerging evidence support β-catenin-independent role of BCL9. Here, we report a binding interaction between BCL9 and PS-727-STAT3. STAT3 is known to have a role in many normal cellular processes, while its activity has been linked to progression of multiple tumor types^34^. For instance, many of STAT3 targets are involved in cellular proliferation (i.e., MYC, CCND1), survival (i.e., BCL2), invasion and metastasis (i.e., ICAM1, MMP2, MMP9, MMP1, MMP10) and encode tumor-promoting cytokines (IL6, IL11 and CXCL12)^35–37^. On the contrary, tumor suppressive properties of STAT3 have also been reported through the regulation of targets such as FOXP3^37^. STAT3 is phosphorylated on tyrosine 705 (YSTAT3705) in response to many growth factors and cytokines. Once phosphorylated on Y705, STAT3 dimerizes, translocates to the nucleus and binds to GAS elements in the promoters of STAT3 target genes^38^. Constitutive Y705STAT3 and PS-727-STAT3 have been observed in many types of cancers including breast cancers^39^. Previous studies have demonstrated that PS-727-STAT3 may be required for its maximal transcriptional activity^38^. Our data demonstrated the formation of a protein complex between BCL9 and PS-727-STAT3 on the enhancer regions of chromatin, while these complexes were significantly less frequent on promoters and de-enriched on the predicted heterochromatin and repressed regions as expected of active regulatory proteins. Enhancers are a class of regulatory DNA elements composed of clusters of transcription factor binding sites that are able to stimulate transcription over distant genomic sequences^40^. Many recent studies have reported the role of enhancer activation in metastatic progression of human cancers^41^. However, only a few studies have reported the potential role of active enhancers in breast cancer progression^42–44^. While, we have demonstrated a significant association between enhancer sequences that are co-enriched with BCL9 and STAT3 and regulation of targets involved in cancer progression, future studies should address whether the predicted enhancers are functionally active by studies such as ATAC-Seq (Assay for Transposase-Accessible Chromatin using sequencing or open chromatin), lentiviral enhancer reporter constructs (i.e., LentiMPRA)^45,46^ and by HiChIP^47^. The role of BCL9/PS-727-STAT3 complexes in activation of enhancers (i.e., formation of enhancer/promoter looping) will need to be confirmed by deletion/mutations of their binding sites followed by enhancer functional assays.

ChIP-exo studies followed by data analysis using ChExMix demonstrated a small median peak-peak distance between BCL9 and PS-727-STAT3 suggestive of a binding interaction. A strong BCL9/PS-727-STAT3 co-occurring peaks were also observed at the predicted binding motifs of other transcription factors including ETS (ELK4 and ELF5), C/EBP family members, AP-1, REL and a large number of zinc-finger containing (YYCCTBCC) transcription factors (~20,000 binding motifs). Unexpectedly, while PS-727-STAT3 samples showed strong binding frequency at STAT3 motifs, BCL9 peaks were not found around STAT3 binding motifs. These data indicate that BCL9/PS-727-STAT3 complexes may associate with chromatin through binding motifs of non-STAT3 transcription factors. Two previous studies also reported that constitutive Y705STAT3 increases the subsequent transcription of an unphosphorylated form of STAT3 referred to as U-STAT3. Consequently, U-STAT3 forms a dimer with transcription factors, such as RELA, AP1, C/EBP, and ETS to induce the transcription of oncogenes such as MET, MRAS, BCL2A1, and RANTES^39,48^. Based on these reports as well as our own data, we propose that BCL9 in a complex with STAT3 may bind to other transcription factors to regulate transcription of oncogenic targets. The majority of these oncogenic targets may not constitute direct STAT3 transcriptional targets.

We have found integrin signaling, in particular integrin β3, in both the RPPA and RNA sequencing studies as one of the top pathways downregulated in BCL9-KD cells compared to non-silencing control cells. This was intriguing since integrin αVβ3 has an established role in cancer progression by promoting cellular invasion^49,50^. We also showed that BCL9 regulation of integrin β3 was independent of the Canonical Wnt pathway activation. ChIP-qPCR studies validated the co-enrichment of BCL9 and STAT3 on two enhancer elements associated with *ITGB3*. Furthermore, we showed that BCL9 transcriptional regulation of integrin β3 played a key role in DCIS invasive progression. An association between integrin expression and cancer progression has been previously established^51^. Our studies further validated these findings since silencing of integrin αVβ3 in our basal type DCIS.COM cells led to in vivo inhibition of invasion. However, SUM225 did not express a high level of integrin β3 like DCIS.COM. While BCL9-KD inhibited DCIS progression in both cell lines, we propose that BCL9 may regulate different sets of target genes in SUM225 to promote invasive progression.

We have also demonstrated that BCL9 transcriptional regulation of MMP16, a canonical Wnt target, played a key role in DCIS progression. In gastric cancer, MMP16 expression was shown to be upregulated by Wnt activation^52^. Similarly, our data indicate that Wnt3A treatment of DCIS cells resulted in increased MMP16 mRNA levels. The effect of MMP16 on cancer progression has been reported in multiple cancers, such as prostate cancer, colon cancer and melanoma, where it promotes migration, invasion, and metastasis^53,54^. Higher levels of MMP16 are detected in cancer tissues compared to normal tissues and they are also associated with poor prognosis^53^.

Our study also revealed a significant colocalization between MMP16 and integrin αVβ3 in patient DCIS and DCIS/IDC tissues. In particular, the co-expression of these two proteins increased in the more advanced stages of DCIS. The only MMP that has been shown to colocalize with integrin αVβ3 is MMP14. In glioma and melanoma cells, MMP14 and integrin αVβ3 colocalized in the cytoplasm as indicated by IF staining^55,56^. MMP14 and integrin αVβ3 complexes are capable of activating MMP2 activity with the assistance of TIMP2 in breast cancer and melanoma cells^55,56^. Comparably, Zhao et al.^57^ discussed the ability of MMP16 to cleave and partially activate MMP2, yet the complete activation requires a low level of TIMP2. Even though we have not tested the function of MMP16 and integrin αvβ3 on MMP2 activity, our data highly suggest that this complex is involved in DCIS progression. Therefore, high co-expression of MMP16 and integrin αvβ3 could serve as an indication of poor prognosis in breast cancer patients and disruption of the binding between these two proteins is a possibility for prevention of DCIS invasive progression.

CA is a natural organic compound isolated from rosemary. Many scientific studies have shown that CA has anti-inflammatory, antioxidant, and anti-carcinogenic activities. Specifically, carnosic acid could inhibit cancer progression by inducing apoptosis in cancer cells through stimulating the production of reactive oxygen species (ROS) and promoting the Caspase-3 signaling pathway^58,59^. Some researchers believe that CA also reduces cancer cell viability through the inactivation of STAT3 signaling and AKT/mTOR pathway^60,61^. In our research, we discovered that CA effectively prevented DCIS invasive progression in a dose dependent manner. In CA-treated mice, we found decreased nuclear expression of BCL9 and β-catenin, which suggests that the anti-cancer effect of carnosic acid could depend on its ability to degrade BCL9 and β-catenin proteins. De la Roche et al.^29^ demonstrated that carnosic acid was able to disrupt the binding between BCL9 and β-catenin in vitro and β-catenin dependent transcription was interrupted in colon cancer cells. In addition, de la Roche et al. observed a degradation of activated β−catenin due to the loss of BCL9 binding and stabilization^29^. A recent study demonstrated that CA binds the ligand-binding domain of the androgen receptor (AR) and degrades AR via ER stress-mediated proteasomal degradative pathway^30^. We speculate that the same mechanism may also promote the degradation of β-catenin and BCL9 in DCIS cells, although further studies are needed to confirm this mechanism.

In conclusion, we have discovered a unique mechanism for BCL9 which is, the formation of BCL9/PS-727-STAT3 complexes on enhancers and subsequent transcription of target genes involved in driving DCIS invasive progression. This binding interaction between STAT3 and BCL9 on enhancers has not been reported previously. We also propose that in breast cancers with aberrant BCL9 expression, BCL9, through the regulation of enhancers may result the expression of multiple oncogenic targets to drive DCIS malignancy (Model: Supplementary Fig. 9). Furthermore, rosemary extract (or carnosic acid) may provide a new therapeutic strategy for prevention of breast cancer by targeting BCL9. Most importantly, these data may have relevance to other cancers that show similar aberrant expression of BCL9 such as bladder, lung, liver, cholangiocarcinoma, and ovarian cancers^15,16^.

## Methods

### Cell culture

LentiX 293T, DCIS.COM, and SUM225 cells were purchased from Asterand, Inc. (Detroit, MI) and were maintained according to the supplier’s guidelines. Both cell lines have been authenticated by genomic profiling and tested as negative for mycoplasma.

### Specimen collection

Patients gave written informed consent for participation in the University of Kansas Medical Center Institutional Review Board–approved study allowing collection of additional biopsies and or surgical tissue for research. Subject recruits included patients undergoing image-guided core-needle biopsy or surgical excision (lumpectomy or mastectomy) due to suspicion of DCIS. All tissue specimens were obtained from individuals following the U.S. Common Rule. In all cases, research specimens were obtained only after acquisition of diagnostic specimens. Following collection, biopsy tissue was placed in preservation media (LiforCell, Lifeblood Medical, Inc.) and stored at 4 °C or on ice until processing, as described previously, to isolate the epithelial and stromal cell components^11^.

### Animals and transplantation

Recipients were 8- to 10-week-old virgin female NOD-SCID IL2Rgamma^null^ (NSG) mice, which were purchased from Jackson Laboratories. Animal experiments were conducted following protocols approved by the University of Kansas School of Medicine Animal Care and Use and Human Subjects Committee. Methods for the MIND model have been previously published^14,62^.

### Immunofluorescence staining (IF)

IF was performed as previously described in ref. ^14^. Antibodies are listed in Supplementary Table 2.

### Western blot analysis and co-immunoprecipitation

Methods have been published previously^14^, and the antibodies used are listed in Supplementary Table 2. All blots shown in the figures were derived from the same experiment and were processed in parallel. Full uncropped images of the blots are shown in Supplementary Fig. 10.

### In vivo invasion studies

Methods have been previously published^14^. In brief, invasive lesions were identified by the lack of a SMA-expressing myoepithelial layer in three consecutive sections and by presence of invasive cells in the surrounding stroma. To determine the number of invasive areas per section, confocal images (×20 magnification) were taken of all invasive lesions and counted. Measurements (i.e., number of invasive areas and number of invasive lesions) for the three sections were averaged to represent each gland.

### Tissue microarray (TMA) analysis

TMAs were constructed from paraffin-embedded, formalin-fixed sections of breast tissue from patients diagnosed with pure DCIS (*n* = 30) or both DCIS and IDC (*n* = 60). Tissue specimens were obtained from individuals enrolled under an IRB approved protocol and following U.S. Common Rule.

### ChIP-EXO

DCIS.COM cells were plated in 15 cm plates. Sixty million cells were collected per replicate, and crosslinking was performed using 1% formaldehyde. Chromatin preparation, immunoprecipitations, and sequencing were carried out by Peconic LLC (State College, PA), using highly specific polyclonal rabbit anti-BCL9 or anti-P(S727)STAT3 (Supplementary Table 2).

### ChIP-EXO peak-calling, merging replicate samples, cross-sample comparison, and peak to gene analysis

ChExMix is a peak-caller designed to work specifically with ChIP-exo data. Assuming that different regulatory complexes can form fundamentally different crosslinking patterns, ChExMix was designed to identify distinct binding subtypes using mixture modeling to combine DNA sequence and the unique distribution of tags around binding sites. Sample replicates were then merged using samtools and ChExMix was re-ran on the merged BAM files to generate consensus peaks for each sample. For cross-sample comparison, sample peaks midpoints were expanded to a 100 bp window centered on the midpoint and intersected with each other. Peaks from each sample were compared against every other peak file to identify the median peak-peak distance in order to identify potential interactions. A low peak-peak median distance is evidence for common occurrence in the sample regulatory region. Peak occurrence relative to their closest refSeq gene was determined.

### Genomic feature enrichment, motif analysis

For transcription start site (TSS) pileup, RNA-seq data for MCF7 generated by the ENCODE project was used to sort refSeq TSS’s by steady-state expression level. The 5’ ends of aligned sequence reads were piled up relative to TSS. A strong gene expression-dependent pattern was observed in the promoters of genes for BCL9 and STAT3-S727ph. In order to better identify potential protein–protein interactions, peaks were intersected with the genomic locations of motifs. Using the published JASPAR motifs, the FIMO software was used to identify all motif occurrences in the hg19 reference genome. Motifs were then filtered to overlap within 100 bp of at least one peak from the various ChIP-exo samples. Tag pileups and composite plots were constructed as previously described.

### Gene ontology enrichment, and genome annotation enrichment

Peaks from merged samples were uploaded to the GREAT webserver using default parameters in order to determine Gene Ontology (GO) enrichment (http://great.stanford.edu/public/html/). For chromatin state annotation, we used the chromatin states predicted by (chromHMM) in HMEC cells (closest to human epithelial mammary available). Peaks from merged samples were compared against predicted regulatory region in HMEC. ChExMix peaks were called on the IgG-negative control in order to determine a background model in this cell line.

### RNA-Seq, RNA isolation, and quality control

Control and BCL9-KD DCIS.COM cells were plated in T75 flasks, one flask per replicate. Total RNA was isolated from each T75 flask using the Qiazol reagent and miRNeasy minikit (Qiagen), according to the manufacturer’s protocol. RNA quantity and quality were measured using nanodrop and agilent. Only when the ratio of the absorbance at 260 nm and 280 nm was between 1.8 and 2.2, the total RNA sample was submitted to Novogene Corporation Inc. (Sacramento, CA) for RNA-seq.

### RNA-Seq data analysis

Downstream analysis was performed using a combination of programs including STAR, HTseq, Cufflink, and our wrapped scripts. Alignments were parsed using Tophat program and differential expressions were determined through DESeq2/edgeR. For reads mapping to the reference genome, reference genome and gene model annotation files were downloaded from genome website browser (NCBI/UCSC/Ensembl) directly. Indexes of the reference genome was built using STAR and paired-end clean reads were aligned to the reference genome using STAR (v2.5). STAR used the method of Maximal Mappable Prefix(MMP), which can generate a precise mapping result for junction reads. To quantify gene expression level, HTSeq v0.6.1 was used to count the read numbers mapped of each gene, and then FPKM of each gene was calculated based on the length of the gene and reads count mapped to this gene. FPKM, Reads Per Kilobase of exon model per Million mapped reads, considers the effect of sequencing depth and gene length for the reads count at the same time, and is currently the most commonly used method for estimating gene expression levels^63^.

For DESeq2 with biological replicates, differential expression analysis between two conditions/groups (two biological replicates per condition) was performed using the DESeq2 R package (2_1.6.3). DESeq2 provide statistical routines for determining differential expression in digital gene expression data using a model based on the negative binomial distribution. The resulting *P*-values were adjusted using the Benjamini and Hochberg’s approach for controlling the False Discovery Rate (FDR). Genes with an adjusted *P*-value < 0.05 found by DESeq2 were assigned as differentially expressed. For edgeR without biological replicates, Prior to differential gene expression analysis, for each sequenced library, the read counts were adjusted by edgeR program package through one scaling normalized factor. Differential expression analysis of two conditions was performed using the edgeR R package (3.16.5). The *P*-values were adjusted using the Benjamini & Hochberg method. Corrected *P*-value of 0.05 and absolute fold-change of 1 were set as the threshold for significantly differential expression. The Venn diagrams were prepared using the function vennDiagram in R based on the gene list for different group.

### Quantitative real-time PCR (qPCR)

RNA was isolated from cells using the Qiagen *RNeasy* Mini Kit (Qiagen 74104) and reverse transcribed to cDNA using superscript III (Invitrogen). Samples and universal human reference RNA (Stratagene) were assayed in triplicate for β-actin, ITGB3, ITGAV, AXIN2, and MMP16 gene expression using specific primer and probe sets and TaqMan chemistry. While primer and probe sequences are proprietary information, assay numbers are provided in Supplementary Table 3.

### Statistical analysis

Statistical comparisons of in vitro studies were conducted using a one-way ANOVA followed by a multiple comparisons test. Statistical comparisons of the migration, invasion assays and flow cytometry data were conducted using a two-sided Student *t*-test. For the MIND xenograft studies, tests are based on estimates from negative binomial mixed effects models. Negative binomial models are used to model the effect of predictors on count data, particularly when the mean and variance cannot be assumed equal. This allows us to account for heterogeneity in the samples. For all comparisons, *P* < 0.05 was considered statistically significant.

### Fluorescence in situ hybridization

FISH procedures were performed on fixed cells on slides, according to manufacturer’s instructions (Empire Genomics). Prior to hybridization, the slides were placed in 70%, 80%, and 100% ethanol solution at room temperature. BCL9 and control probes were obtained from Empire genomics (Cat# BCL9-20-RE and CHR01-10-GR, respectively). Co-denaturation was performed at 73 °C for 5 min, followed by an overnight hybridization at 37 °C. The slides were then washed with 0.4× SSC/0.3% NP-40 at 73 °C for 2 min, then in 2× SSC/0.1% NP-40 at RT for 1 min. The cells were counter-stained with DAPI.

### Plasmids, transfection, and luciferase reporter assay

PCDH-BCL9 (BCL9-OE), PLKO.1-BCL9-shRNA (BCL9-KD) (CCTCTGTTGAATATCCCTGGAA), and PLKO.1-non-silencing control (Control) were acquired from Dr. Carrasco^31^. For additional shRNA knockdown procedures, pGIPZ Human MMP16 shRNA (MMP16 KD) (Dharmacon #V2LHS_198095), pGIPZ Human ITGAV shRNA (Dharmacon # V2LHS_133468), pGIPZ Human ITGB3 shRNA (Dharmacon #V2LHS_77099), and pGIPZ-non-silencing control (Control) (Dharmacon # RHS4346) were obtained. For STAT3 signaling activity, Cignal STAT3 Reporter (luc) kit (Qiagen # CCS-9028L) was used. Plasmid constructs: Constitutively active STAT3 overexpression, EF.STAT3C.Ubc.GFP (Addgene plasmid 24983) was provided by Linzhao Cheng via Addgene. pLEGFP-Y705F-STAT3 (Addgene plasmid # 71445; http://n2t.net/addgene:71445; RRID:Addgene_71445), pLEGFP-WT-STAT3 (Addgene plasmid # 71450; http://n2t.net/addgene:71450; RRID:Addgene_71450), and pLEGFP-S727A-STAT3 (Addgene plasmid # 71446; http://n2t.net/addgene:71446; RRID:Addgene_71446) were gifts from George Stark. Transfection: DCIS.COM and SUM225 cells were transfected with electroporation using Amaxa^TM^ Cell line Nucleofector kit V (Lonza #VCA-1003), while HEK293T cells were transfected using Lipofectamine 2000 reagent (Invitrogen #11668-027) according to manufacturer’s protocols. Luciferase assays were performed using the Dual-Luciferase^®^ Reporter Assay System (Promega #E1910).

### Lentivirus production

Glycerol stocks of shRNA-based lentiviral plasmids cultured with 100 μg/ml of ampicillin (Amresco # 0339) and plasmids were purified using HiSpeed Plasmid midi kit (Qiagen #12643). Preparation of viral particles was performed by co-transfecting individual vectors (10 μg), packaging plasmid pCMV-dR8.2 (contains Gag, Pol, Rev, and Tat) (Addgene plasmid 8455; 5 μg), and the envelope plasmid pCMV-VSVG (Addgene plasmid 8454; 5 μg) in HEK293T cells. Both plasmids were acquired from Robert Weinberg via Addgene^64^. Transfection was performed in a 10 cm plate, at 75% confluency, using Lipofectamine 2000 transfection reagent (Invitrogen #11668-027) in antibiotic free Opti-MEM media (Invitrogen #51985-034) following manufacturer’s protocol. After 48, 72, and 96 h of transduction, media was collected, pooled and subjected to ultracentrifugation at 80,000 × *g* for 2 h (Beckman Coulter, Optima L-100 XP, 70Ti rotor) at room temperature. Pellets of the concentrated viral particles were resuspended in 250 μl of DMEM (GIBCO #21063029) and stored in aliquots at −80 °C until further use. Lentiviral titers were measured using Lenti-X^TM^ p24 Rapid Titer kit (Clontech #632200). Transduction was performed at an MOI of 5 and 20 for DCIS.COM and SUM225 cells, respectively, and both lines continued to grow in the presence of puromycin (Thermo Scientific #100552). For all experiments, only the transduced cells from up to 5 passages were used.

### FACS analysis

Cells were stained at a final dilution of 1∶100 for 30 min on ice followed by washes in Hanks’ Balanced Salt Solution (Invitrogen #24020-117) containing 2% fetal bovine serum. Antibodies used are listed in S.Table 2. FACS and data analysis were performed using the BD LSR II flow cytometer and FlowJo software (Tree Star).

### MMP activity assays

DCIS.COM and SUM225 cells were seeded at 2 × 10^6^ cells/ well in 6-well plates, cell culture media was changed into DCIS.COM or SUM225 media supplemented with 2% FBS and collected after 24 h. MMP activity assays were performed using SensoLyte^®^ 520 Generic MMP Assay Kit*Fluorimetric* (AnaSpec, # AS-71158) following the manufacturer’s protocol.

### Chromatin immunoprecipitation (ChIP) assays

ChIP assays were performed using Active Motif’s ChIP-IT^®^ Express Chromatin Immunoprecipitation Kit (Active Motif 53008) following the manufacturer’s protocol with the following specifications: DCIS.COM cells (1.5 × 10^7^ per plate) were plated on 150 mm plates and allowed to attach overnight. Following cell collection and fixation, sonication was performed in a Bioruptor^®^ Sonicator (Diagenode). Immunoprecipitation was performed overnight with 100 μl of chromatin and 2 ug rabbit anti-BCL9, rabbit anti-STAT3 antibodies or IgG control. Primers for qPCR were designed to encompass the putative STAT3 binding site within the *ITGB3* gene (Supplementary Table 3). DNA purified from ChIP samples and input DNA, along with a standard curve made of serial dilutions of input DNA were subject to qPCR on Applied Biosytem’s *StepOnePlus*™ Real-Time PCR System. ChIP qPCR results were normalized to input DNA and presented as fold increase over IgG control.

### Reverse phase protein array (RPPA)

RPPA lysis buffer, protease inhibitors, phosphatase inhibitors, and SDS Sample Buffer were provided by Cancer Prevention and Research Institute of Texas (CPRIT) Cancer Proteomic and Metabolomic Core Facility at Baylor College of Medicine (https://www.bcm.edu/centers/cancercenter/research/shared-resources/cprit-cancer-proteomics-and-metabolomics/reverse-phase-proteinarray). To prepare cell lysates, cells were harvested by trypsinization, washed twice with cold PBS and centrifuged at 400 × *g* for 5 min. The pellet (5 × 10^6^ cells) was resuspended in 300 μl RPPA working solution (composed of 1 ml protease Inhibitors, 1 ml phosphatase inhibitors, and 3 ml RPPA lysis buffer). Proteins were incubated on ice and vortexed every 10 min for 30 min. Lysates were centrifuged at 14,000 × *g* for 15 min at 4 °C, and the supernatant was transferred to a fresh tube. Pierce^TM^ BCA Protein Assay Kit (Fisher Scientific #23225) was used to determine protein concentration.

The lysates were diluted into 0.5 mg/ml of total protein in SDS sample buffer and denatured on the same day. RPPA analyses were carried out as described previously with miner modifications^65,66^. Specifically, the Aushon 2470 Arrayer (Aushon BioSystems, Billerica, MA) was used to spot samples and control lysates onto nitrocellulose-coated slides (Grace Bio-labs, Bend, OR) which were probed with a set of 224 antibodies against total proteins and phosphoproteins using an automated slide stainer Autolink 48 (Dako, Santa Clara, CA). Each slide was incubated with one specific primary antibody and a negative control slide was incubated with antibody diluent without any primary antibody. Primary antibody binding was detected using a biotinylated secondary antibody followed by streptavidin-conjugated IRDye680 fluorophore (LI-COR Biosciences, Lincoln, NE). Total protein content of each spotted lysate was assessed by fluorescent staining with Sypro Ruby Protein Blot Stain (Molecular Probes, Eugene, OR).

Fluorescence-labeled slides were scanned on a GenePix 4400 AL scanner, along with accompanying negative control slides, at an appropriate PMT to obtain optimal signal. The images were analyzed with GenePix Pro 7.0 (Molecular Devices). Total fluorescence signal intensities of each spot were obtained after subtraction of the local background signal for each slide and were then normalized for variation in total protein, background and non-specific labeling using a group-based normalization method as described^65^.

### IPA canonical pathway analysis

Canonical pathways analysis identified the pathways from the Ingenuity Pathway Analysis library of canonical pathways that were most significant to the dataset^17^. Molecules from the dataset that met the −log *P*-value cutoff higher than 1.3 and were associated with a canonical pathway in the Ingenuity Knowledge Base were considered for the analysis. The significance of the association between the dataset and the canonical pathway was measured in two ways: (1) A ratio of the number of molecules from the dataset that map to the pathway divided by the total number of molecules that map to the canonical pathway is displayed; and (2) A right-tailed Fisher’s Exact Test was used to calculate a *p*-value determining the probability that the association between the genes in the dataset and the canonical pathway is explained by chance alone.

### Proximity ligation assays

Duolink^®^ In Situ Red Starter Kit Mouse/Rabbit (Sigma-Aldrich # DUO92101-1KT) was used. DCIS.COM cells were fixed and permeabilized with 0.2% Triton X-100 at room temperature and then blocked for 1 h at 37 °C using Duolink^®^ PLA Blocking Buffer. Cells were then incubated with mouse anti-β-catenin and rabbit anti-BCL9 primary antibodies (right), or mouse anti-P(S727) STAT3 and rabbit anti-BCL9 primary antibodies (Left). Technical negative controls included incubation with each primary antibody separately and no primary antibody. After washing, Duolink^®^ PLA were performed according to the kit’s protocol.

### Reporting summary

Further information on research design is available in the Nature Research Reporting Summary linked to this article.

## Supplementary information

Supplementary information

Reporting Summary Checklist

Supplementary data 1

Supplementary data 2

## Data Availability

All RNA sequencing data have been deposited in the Gene Expression Omnibus with accession https://identifiers.org/geo:GSE143790^67^. All Chip-Exo data have been deposited in the Gene Expression Omnibus with accession https://identifiers.org/geo:GSE143313^68^. RPPA data are openly available at *figshare* under 10.6084/m9.figshare.11877411^69^, in the file called ‘Supplementary Fig. 3-RPPA.xlsx’All data used to generate the figures in this manuscript and the Supplementary Information are also available in Excel spreadsheets at the *figshare* data record^69^.
