# Supplementary material for: BCL9/STAT3 regulation of transcriptional enhancer networks promote DCIS progression
Source: NPJ Breast Cancer. 2020 Apr 24;6:12. doi: 10.1038/s41523-020-0157-z (PMC7181646; doi:10.1038/s41523-020-0157-z)
Supplement: Supplementary file 1 — Supplementary information [file 41523_2020_157_MOESM1_ESM.pdf]

SUPPLEMENTARY INFORMATION

BCL9/STAT3 Regulation of Transcriptional Enhancer Networks Promote DCIS Progression

**Authors and affiliations.** Hanan S. Elsarraj[1], Yan Hong[1], Darlene Limback[1], Ruonan Zhao[1], Jenna Berger[2], Stephanie C. Bishop[3], Aria Sabbagh[4], Linzi Oppenheimer[1], Haleigh E. Harper[5], Anna Tsimelzon[6], Shixia Huang[7], Susan G. Hilsenbeck[8], Dean P. Edwards[7], Joseph Fontes[9], Fang Fan[1], Rashna Madan[1], Ben Fangman[5], Ashley Ellis[5], Ossama Tawfik[10], Diane L. Persons[1], Timothy Fields[1], Andrew K. Godwin[1], Christy R. Hagan[9], Katherine Swenson-Fields[11], Cristian Coarfa[7], Jeffrey Thompson[12] and Fariba Behbod[1*]

# Supplementary Figures and Figure legends:

Supplementary Figure 1

a

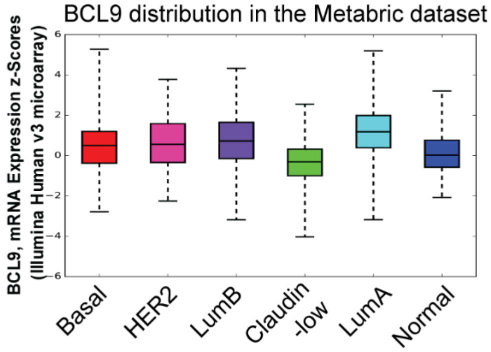


b

| Subtype | Basal | Her2 | LumA | LumB | Normal | Claudin-low |
|---|---|---|---|---|---|---|
| Basal | | 0.610 | *10.369 | *1.674 | *2.106 | *10.734 |
| Her2 | | | *7.022 | 0.465 | *3.850 | *14.723 |
| LumA | | | | *8.533 | *19.246 | *49.094 |
| LumB | | | | | *6.977 | *25.543 |
| Normal | | | | | | *3.978 |
| Claudin-low | | | | | | |

-log10(p-value) 0 10 20 30 40 50

c

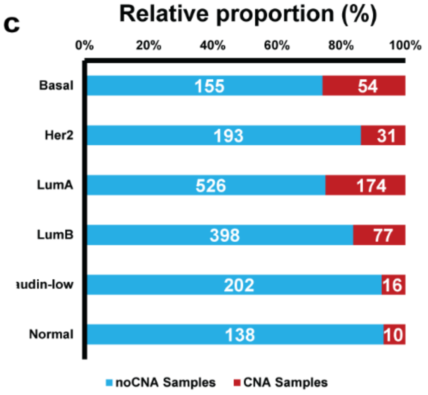


d

| Subtype | Basal | Her2 | LumA | LumB | Normal | Claudin-low |
|---|---|---|---|---|---|---|
| Basal | | *2.630 | 0.105 | *2.368 | *5.623 | *6.700 |
| Her2 | | | *3.367 | 0.361 | *1.382 | *1.513 |
| LumA | | | | *3.428 | *6.811 | *8.542 |
| LumB | | | | | *2.561 | *2.942 |
| Normal | | | | | | 0.000 |
| Claudin-low | | | | | | |

-log10(p-value) 0 2 4 6 8 10

e

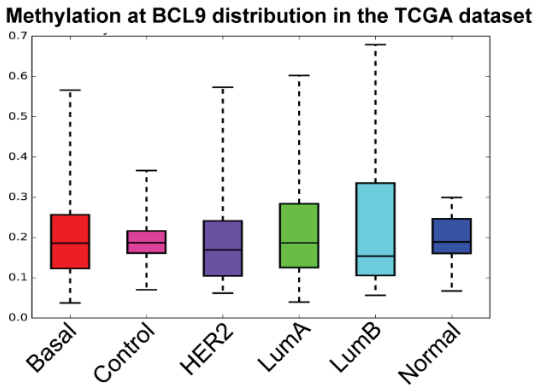


f

| Subtype | Basal | Control | Her2 | LumA | LumB | Normal |
|---|---|---|---|---|---|---|
| Basal | | 0.33 | 0.26 | 0.56 | 0.85 | 0.03 |
| Control | | | 0.07 | *2.14 | *1.72 | 0.22 |
| Her2 | | | | 0.81 | 1.08 | 0.21 |
| LumA | | | | | 0.36 | 0.52 |
| LumB | | | | | | 0.81 |
| Normal | | | | | | |

-log10(p-value) 0 1 2 3

g

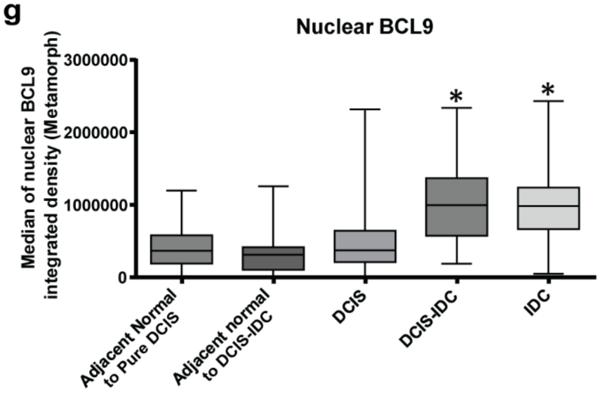

**Supplementary Figure 1: *BCL9* genomic amplification and mRNA upregulation in Luminal A, B and Basal-like breast cancers.** Analysis of Breast cancer METABRIC Data including 2509 cases (a-e):

(a) Distribution of BCL9 mRNA expression in 6 breast cancer subtypes presented as Z-scores.

(b) Pairwise comparison of mRNA expression using two-sided t-test with unequal variance expressed as –log10 (p-value).

(c and d) Fraction of samples with copy number alterations (c) and computed pairwise Fisher test for CNA incidence expressed as –log10(p-value) (d).

(e and f) DNA methylation distribution at the *BCL9* promoter region in different breast cancer subtypes (e) and their computed pairwise differences expressed as –log10 (p-value) (f); Methylation data was obtained from The Cancer Genome Atlas (TCGA) dataset.

(g) BCL9 nuclear expression was examined in 60 cases of DCIS with associated IDC and 30 pure DCIS cases. Representative box plots of median nuclear BCL9 integrated density are shown. IF was performed and quantified in adjacent normal tissue to pure DCIS, adjacent normal to DCIS with associated IDC, pure DCIS cases, DCIS component and IDC component in DCIS/IDC cases. Data were analyzed using paired T-test multi-group comparison (* represent statistically significant difference; $P<0.001$). The box plots represent means and the bars represent standard error from the mean

Supplementary Figure 2

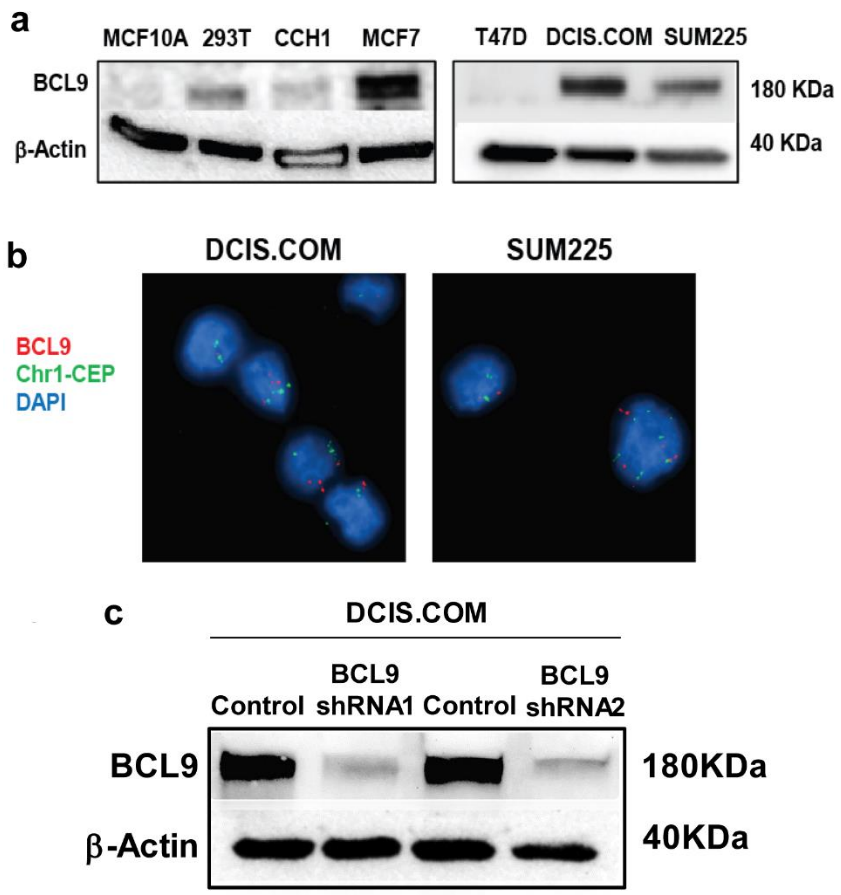


**Supplementary Figure 2. *BCL9* protein expression and genomic amplification in breast cancer cell lines.**

(a) Western blot analysis of BCL9 protein expressing using anti-BCL9 antibody in five breast cancer cell lines: CCH1, MCF7, T47D, DCIS.COM, and SUM225 as well as 293T and MCF10A cells. Anti-β-actin was used as a loading control.

(b) Fluorescence in situ hybridization (FISH) in DCIS.COM and SUM225 cells to identify BCL9 amplification. Chromosome 1 control probe hybridized to the centromeric region of chromosome 1 (Chr1-CEP) and it is labelled with Green 5-Fluorescein. *BCL9* probe is labelled with 5-Rox-dUTP, which appears red. All nuclei are counterstained with DAPI to produce the blue background.

(c) Western blot analysis of DCIS.COM cells transduced with two different BCL9 shRNAs. showing successful BCL9 knockdown.

Supplementary Figure 3

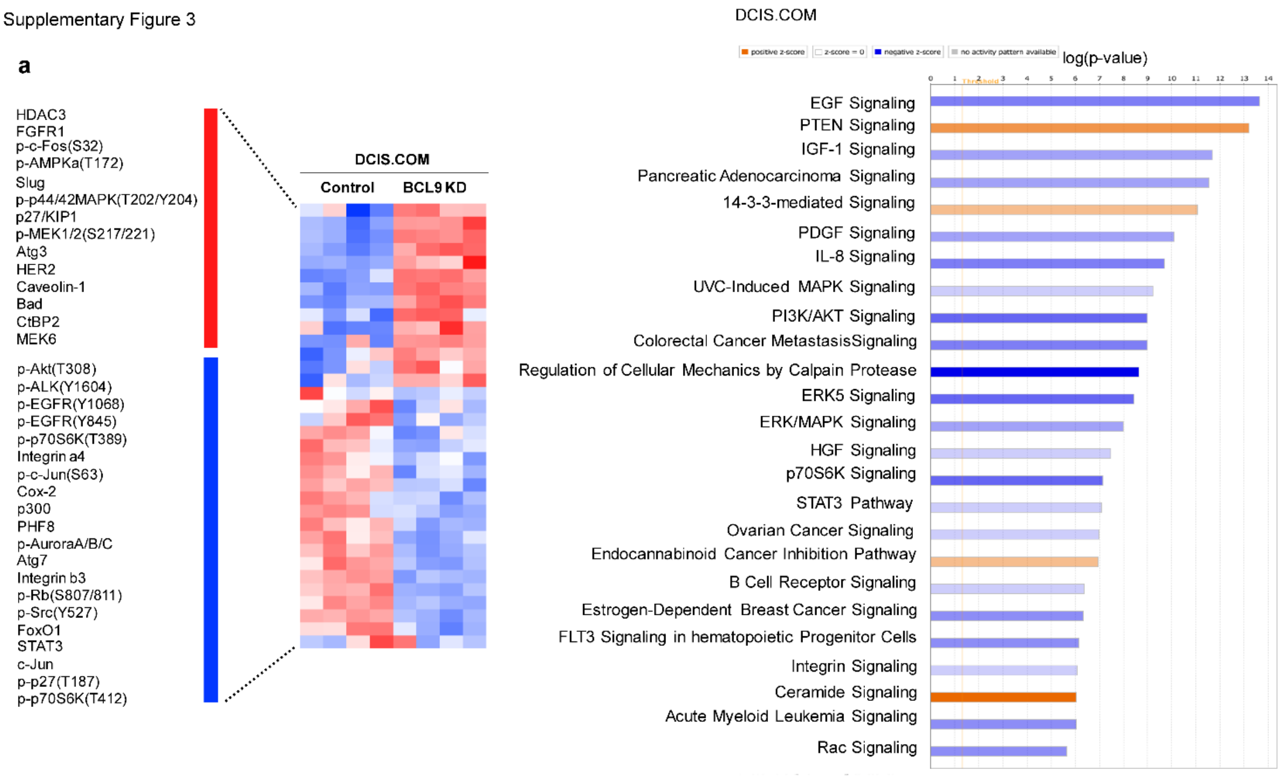


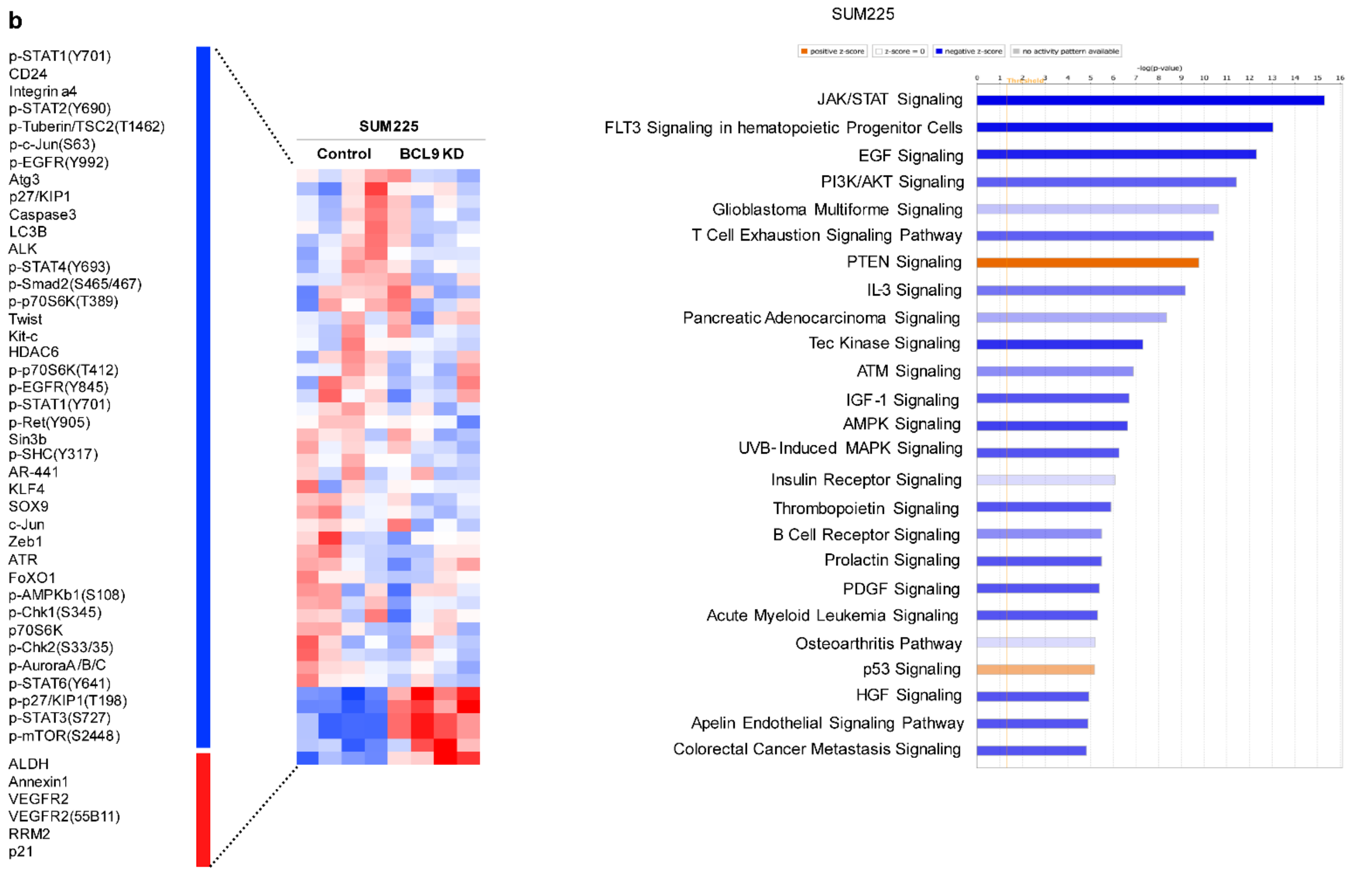

**Supplementary Figure 3. BCL9 regulation of STAT3 targets and relevant to breast cancer.**

Reverse Phase Protein Array (RPPA) was performed on DCIS cell lines with BCL9 KD and control.
(a-b: Left Panels) Heat maps of differentially expressed proteins in BCL9-KD compared to control in DCIS.COM (a) and SUM225 (b).
(a-b: Right Panels) Ingenuity pathway analysis (IPA) of the differentially expressed proteins in DCIS.COM (a) and SUM225 (b) BCL9 KD cells compared to control revealed downregulation of a number of STAT3 regulated signaling pathways with relevance to breast cancer.

Supplementary Figure 4

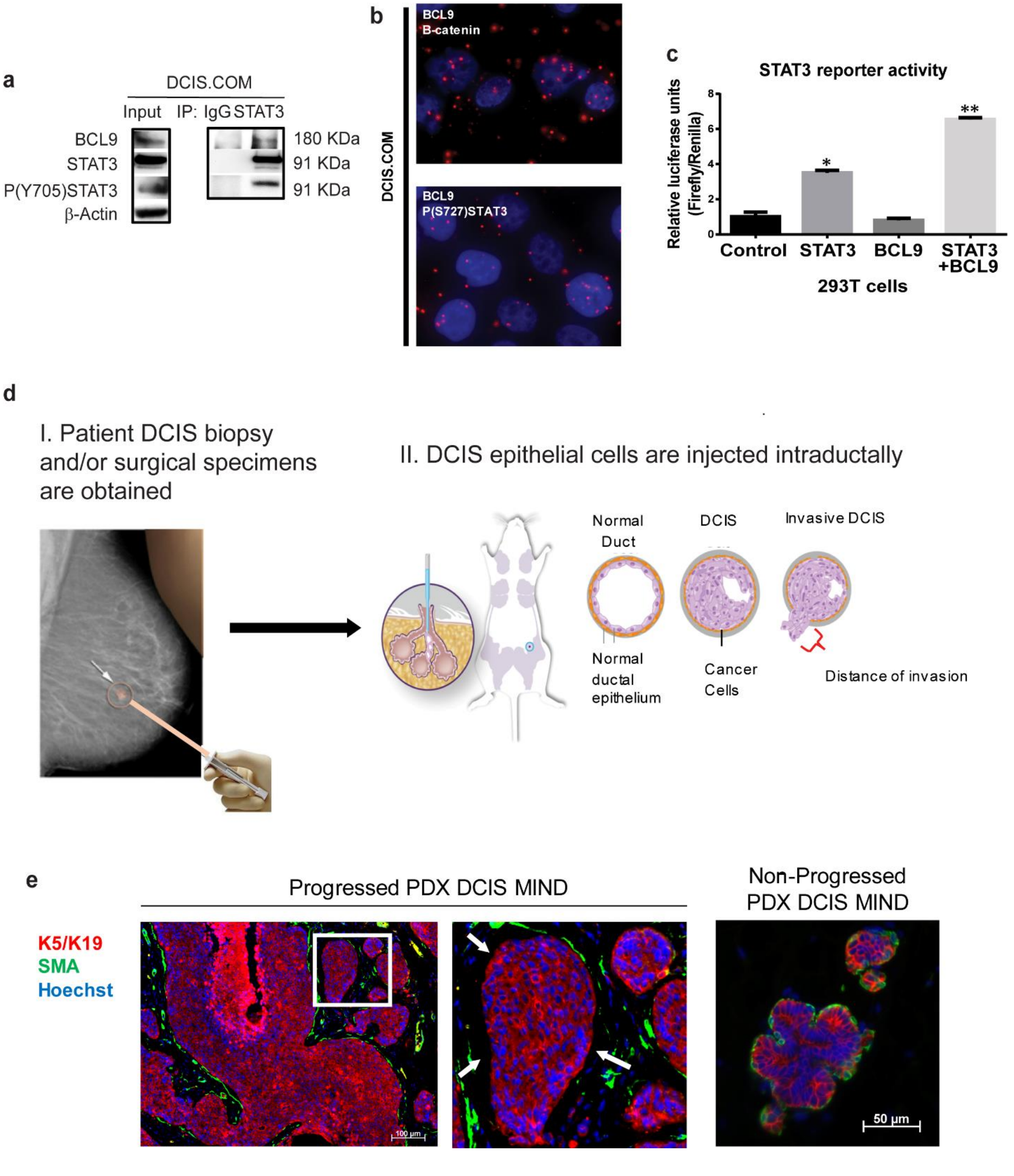

**Supplementary Figure 4. BCL9/STAT3 protein interactions may regulate STAT3 transcriptional activity.**

(a) IP was performed using anti-STAT3 antibody, followed by western blot analysis using anti-STAT3, anti-BCL9 and anti-P(Y705) STAT3 and control IgG antibodies.

(b) PLA using Duolink® PLA kit on DCIS.COM cells using anti-β-catenin and anti-BCL9 antibodies (top), or anti-P(S727) STAT3 and anti-BCL9 antibodies (bottom). PLA signals are identified by fluorescence microscopy as red discrete spots.

(c) STAT3 reporter activity in 293T control, 293T transduced with constitutively active STAT3 (CA-STAT3), BCL9, both CA-STAT3 and BCL9 constructs.

(d) An illustration of the DCIS MIND model.

(e) Representative IF staining with human specific keratins 5 and 19 (red) and SMA (green) in a progressed and a non-progressed PDX DCIS MIND xenografts. White arrows point to areas of SMA loss around the invasive lesions.

Supplementary Figure 5

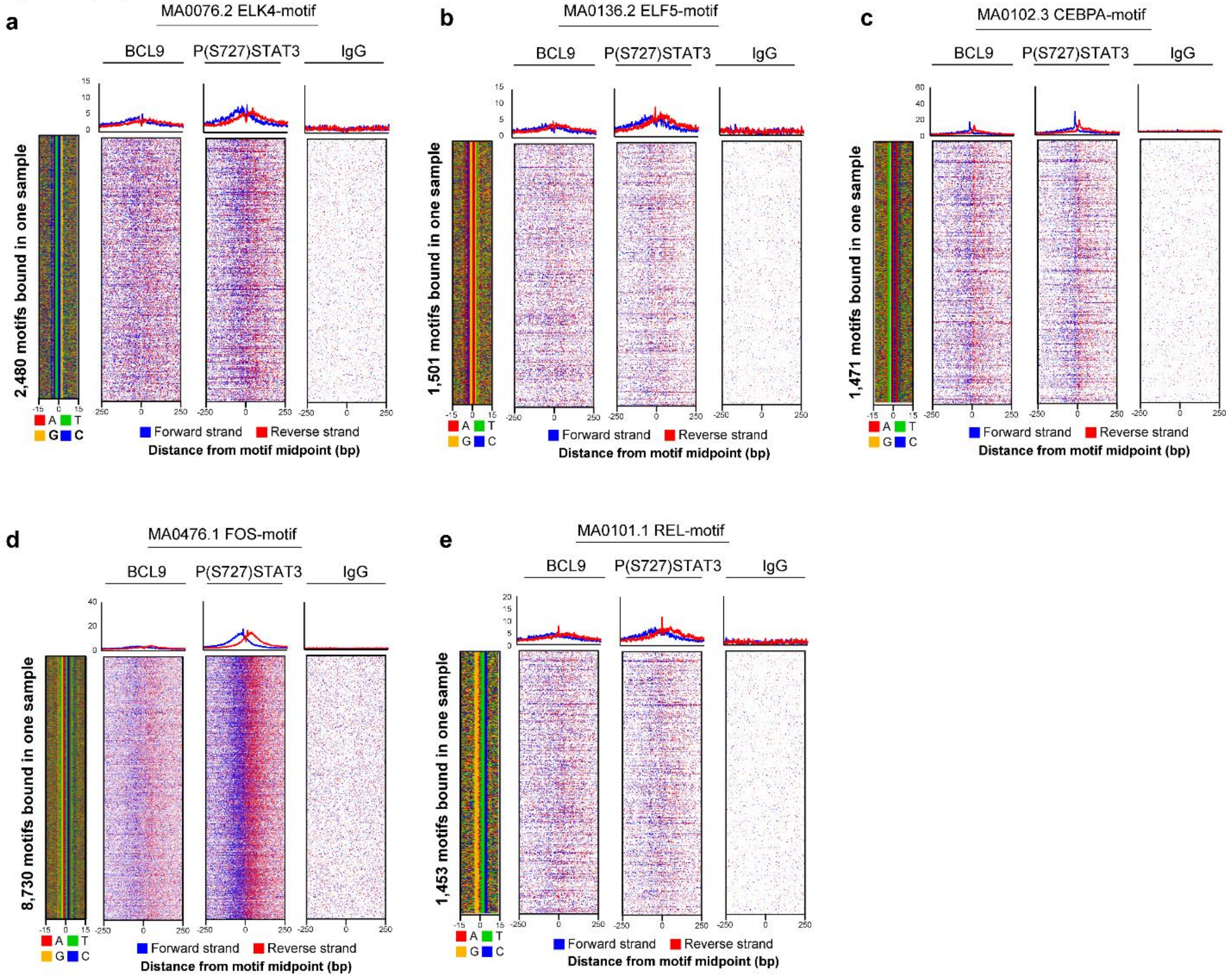


**Supplementary Figure 5. BCL9 and P(S727) STAT3 co-occurring peaks at common transcription factor binding motifs.** Motif analysis found a significant number of BCL9/STAT3 co-occurring peaks near transcription factor binding motifs of ETS (ELK4 and ELF5) (a-b), C/EBP (c), FOS (d) and REL (e).

Supplementary Figure 6

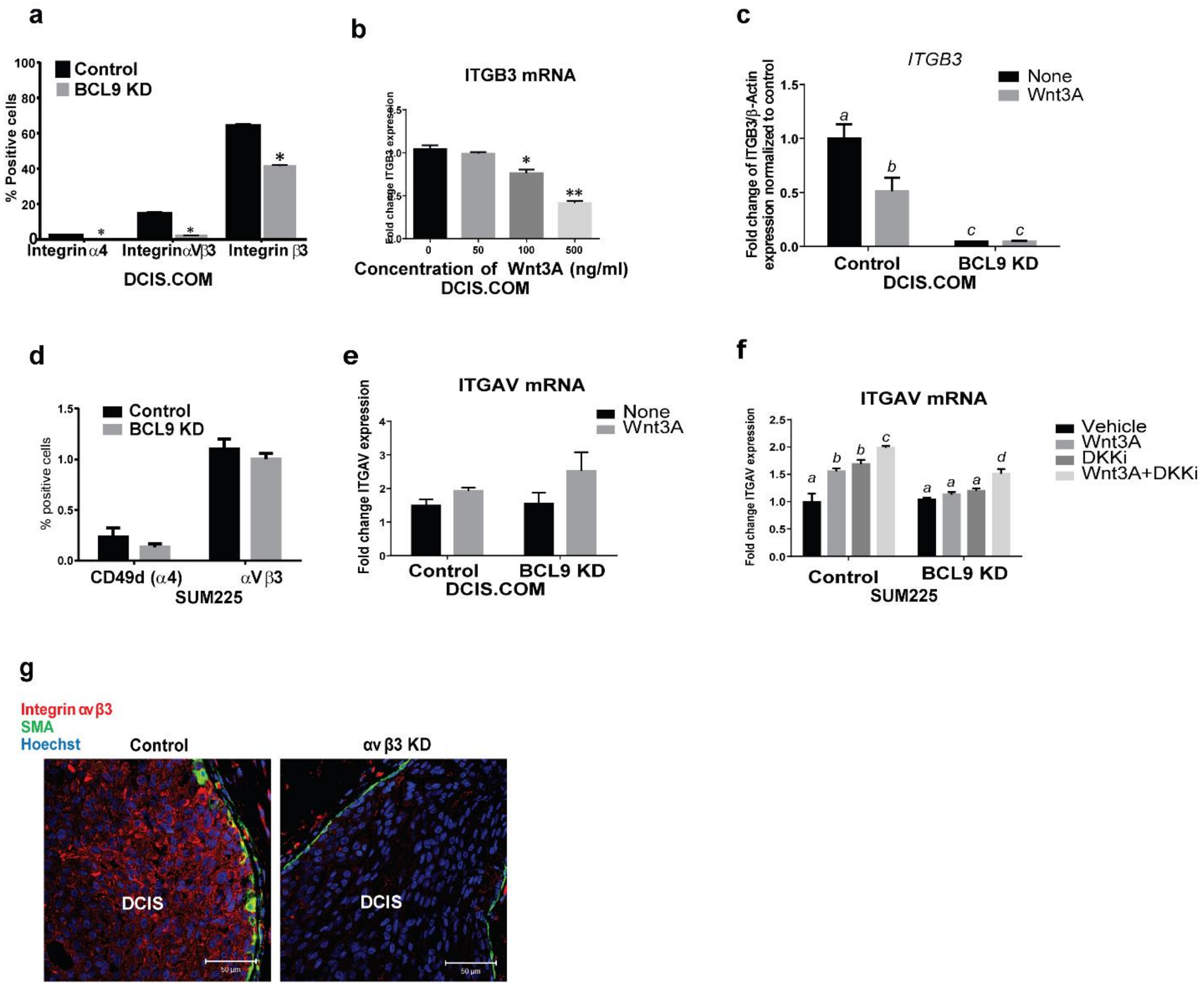


**Supplementary Figure 6. BCL9 regulation of integrin αvβ3 in DCIS cell lines SUM225 and DCIS.COM.**

(a) Bar graphs represent cell surface expression of integrins α4, αVβ3 and β3 in control and BCL9 KD DCIS.COM cells by flow cytometry.

(b) Fold change ITGB3 mRNA expression in DCIS.COM cells in response to increasing concentrations of Wnt3A (50, 100, and 500 ng/ml).

(c) QPCR showing fold change in expression of integrin β3 (*ITGB3*) mRNA levels in cells treated with 500 ng/ml Wnt3a in control and BCL9 KD relative to control. β-Actin was used as normalizing control.

(d) Bar graphs represent surface expression of integrins α4, and αVβ3 in control and BCL9 KD SUM225 cells assessed by flow cytometry.

(e-f) QPCR showing fold change in ITGAV mRNA levels in BCL9 KD relative to control in DCIS.COM and SUM225 cells. Cells were treated with either 500 ng/ml Wnt3A or vehicle control

for DCIS.COM and vehicle control, DKK inhibitor 5uM, 500ng/ml Wnt3A or combined Wnt3A and DKK inhibitor in SUM225.
(a-f) Data were analyzed using unpaired T-test multi-group comparison (* and letters represent statistically significant difference; $P<0.05$; n=3 replicates per group).
(g) Representative IF images of DCIS.COM control and αVβ3 KD MIND xenografts stained with integrin αVβ3 (red), SMA (green), and Hoechst (blue) showing successful knockdown of αVβ3.

Supplementary Figure 7

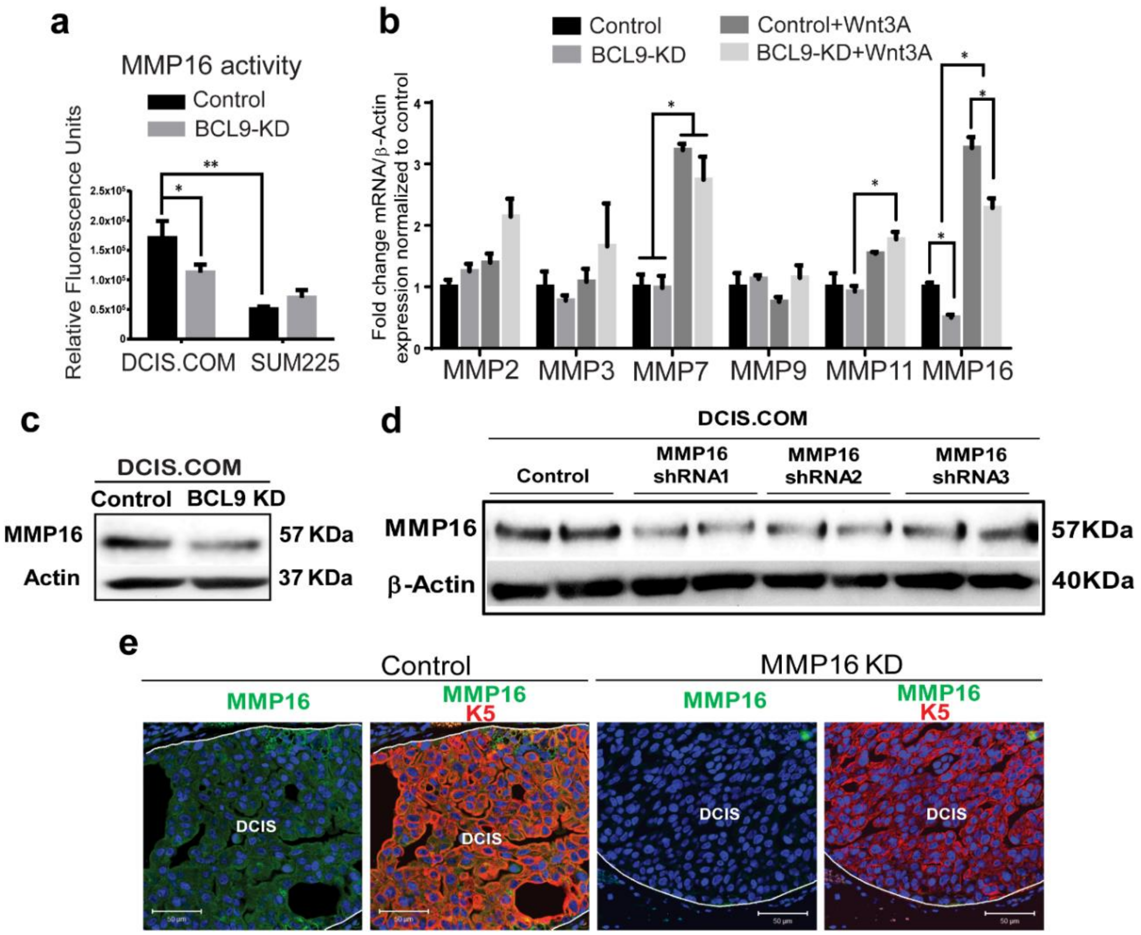


f

| Subtype | Adjacent normal Pure DCIS | Adjacent normal DCIS with IDC | Pure DCIS | DCIS with IDC | IDC |
|---|---|---|---|---|---|
| Adjacent normal Pure DCIS | | 0.372 | *> 4.000 | *> 4.000 | *> 4.000 |
| Adjacent normal DCIS with IDC | | | *> 4.000 | *> 4.000 | *> 4.000 |
| Pure DCIS | | | | 1.258 | *4.000 |
| DCIS with IDC | | | | | *2.958 |
| IDC | | | | | |

-log10(p-value) | 0 | 1 | 2 | 3 | >4

**Supplementary Figure 7. BCL9 regulation of MMP16 and MMP16/integrin αvβ3 co-expression in DCIS.**

(a) Total MMP activity of nine major MMPs measured by fluorescence intensity at Ex/Em=490 nm/520 nm in DCIS.COM and SUM225 control and BCL9 KD cells (n=4).

(b) Fold change of MMP2, MMP3, MMP7, MMP9, MMP11, and MMP16 mRNA levels in BCL9 KD relative to control DCIS.COM cells. Cells were treated with Wnt3a at 500 ng/ml or vehicle control. β-Actin mRNA levels were used for normalization.

(a-b) Data were analyzed using unpaired T-test multi-group comparison (*represent statistically significant difference; $P<0.05$; $n=3$ replicates per group).
(c) Western blot analysis of MMP16 level in DCIS.COM non-silencing (control) and BCL9 KD. β-actin is used as a loading control.
(d) Western blot analysis of DCIS.COM cells transduced with three different MMP16 shRNAs, showing successful knockdown of MMP16. MMP16 shRNA1 was selected for our *in vivo* studies.
(e) Representative IF images of MMP16 KD and control DCIS.COM MIND xenografts stained with MMP16 (green), K5 (red), and Hoechst (blue). Scale bar=50 μm.
(f) Pairwise comparison of differences in % αVβ3-MMP16 co-expression in DCIS tissue samples using two-sided t-test with unequal variance expressed as –log10 (p-value).

Supplementary Figure 8

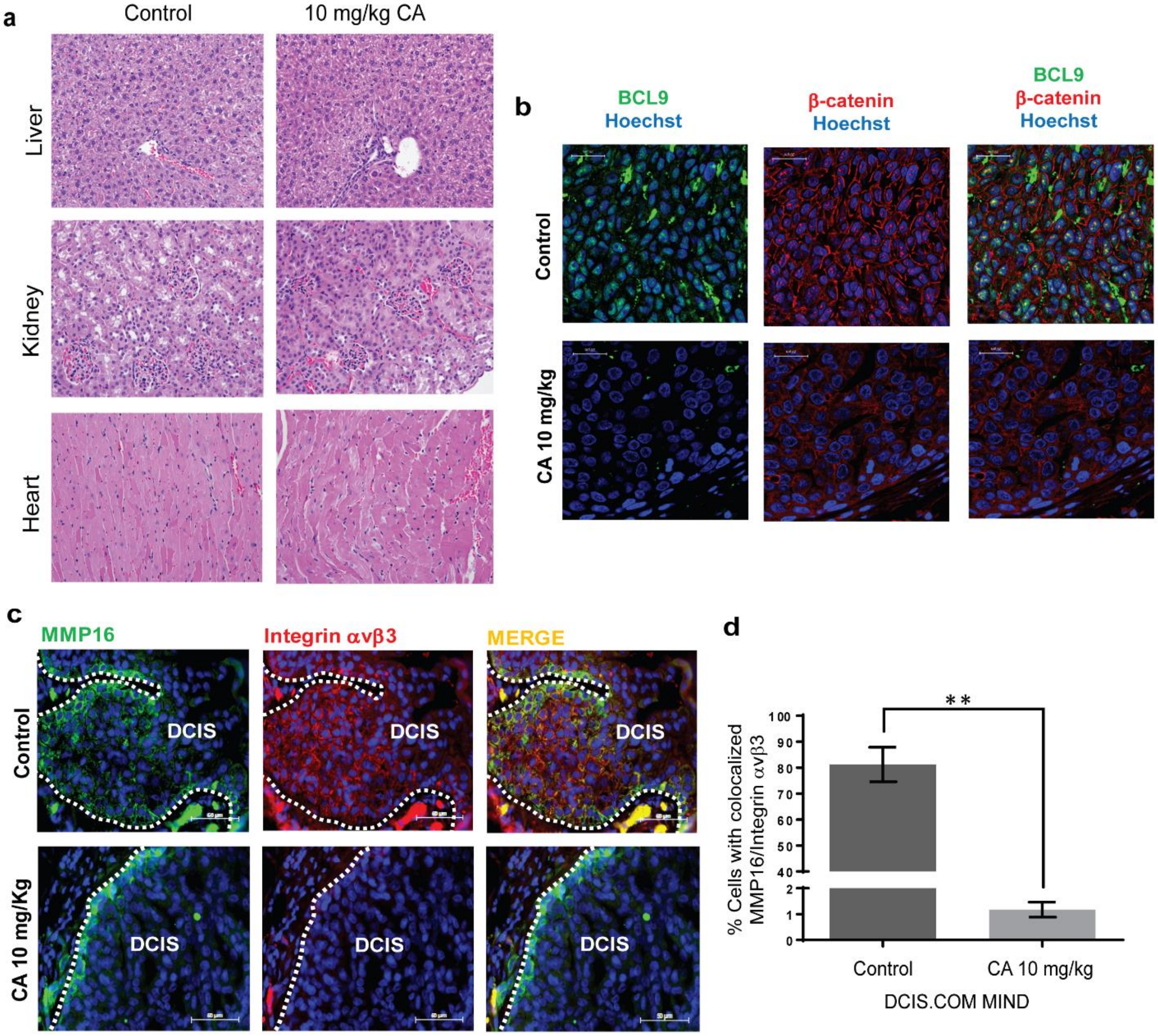


**Supplementary Figure 8a. CA administration did not result in any organ toxicity.** Representative hematoxylin and eosin images of liver, kidney, and heart from mice treated with the maximum dose of 10 mg/kg CA and vehicle control (1% DMSO in olive oil) treated mice.

**Supplementary Figure 8b. CA administration was associated with a significant loss of nuclear BCL9 and β-catenin protein expression in DCIS.COM cells.** IF staining of β-catenin (red), BCL9 (green), and Hoechst in control and CA treated DCIS.COM MIND xenografts showing decreased intensity of nuclear β-catenin and BCL9 in the DCIS cells in CA treated xenografts.

**Supplementary Figure 8c-d. CA administration was associated with a significant loss in the expression of MMP16 and integrin αVβ3 as well as their co-localization. (C)** Representative IF images of DCIS.COM vehicle control and CA 10 mg/kg treated MIND xenografts stained with MMP16 (green), integrin αVβ3 (red), and Hoechst (blue) show colocalization of MMP16 and integrin αVβ3 (yellow areas) in DCIS lesions in control (top row) and reduced expression in CA 10 mg/kg treated animals (bottom row). Scale bars=50 μm. **(D)** Bar graph represents % αVβ3-MMP16 co-localization in control and CA 10 mg/kg treated MIND xenografts. The bars represent mean and standard error of the mean. Data were analyzed using unpaired two-tailed T-test comparison (** represents statistically significant difference; $P<0.01$; n=3 replicates per group).

Supplementary Figure 9

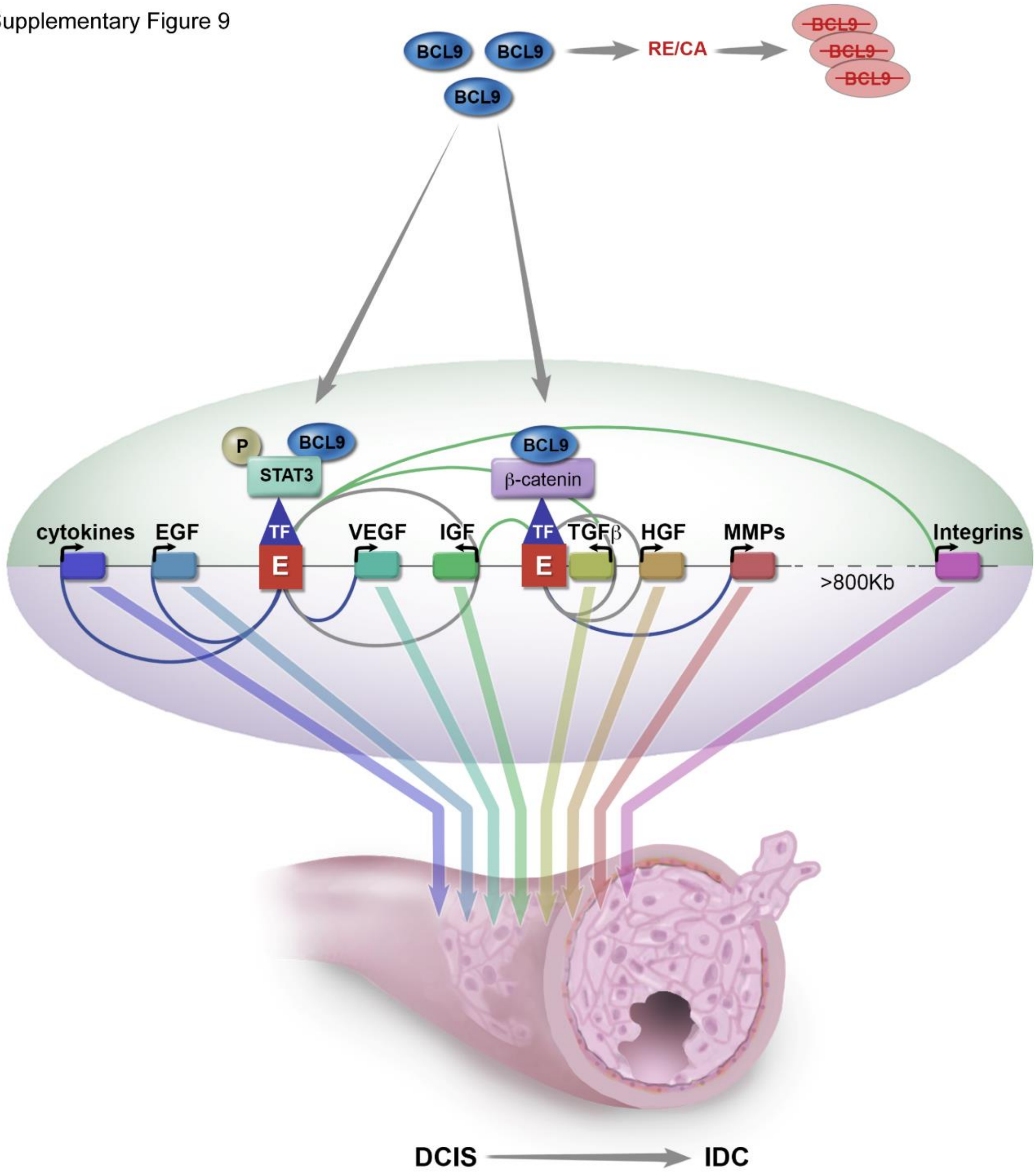


**Supplementary Figure 9. Hypothetical model of BCL9 induced DCIS malignancy.** BCL9 in a complex with STAT3 and/or β−catenin on enhancers drive the expression of growth factors (EGF, IGF, HGF, TGFβ), immune regulatory cytokines as well as mediators of cellular migration and invasion (MMPs and Integrins) to drive DCIS invasive progression. As such, targeting enhancer regulatory networks (i.e, BCL9 targeting by RE/CA) may provide a therapeutic strategy for prevention of breast cancer progression.

**Supplementary Figure 10**. Full uncropped images of blots shown in the paper (Figure 1a, Supplementary Figure 2a and c, Supplementary Figure 4a, and Supplementary Figure 7c and d) including molecular weight markers at the right in KDa when possible. *We were not able to locate the full blot image for STAT3 and b-actin in Figure 1 a.* Predicted molecular weights for BCL9, STAT3, b-catenin, MMP16 and β-Actin are 180 KDa, 86-91 KDa, 92 KDa, 57 KDa, and 37 KDa, respectively.

**Figure 1a**

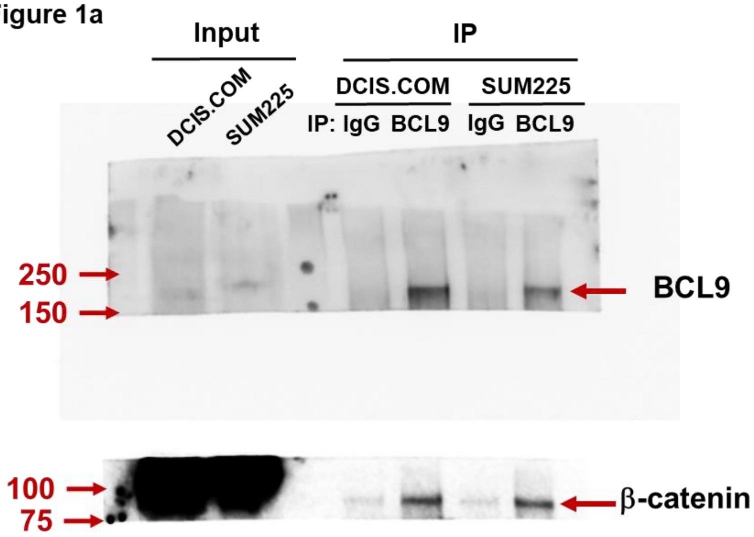

Supplementary Figure 2a

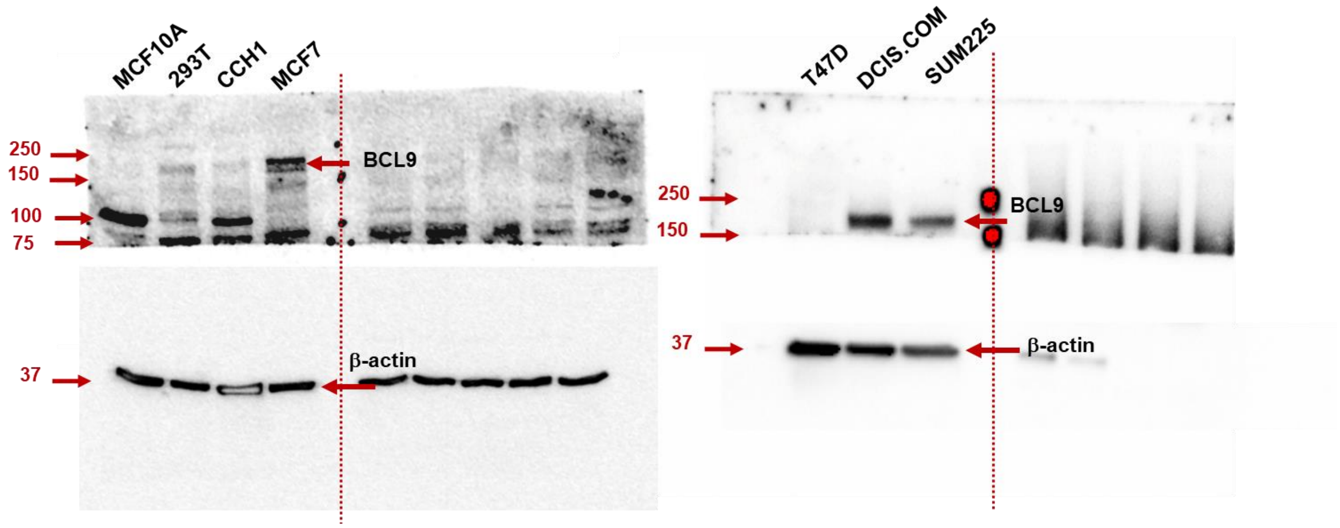


Supplementary Figure 2c

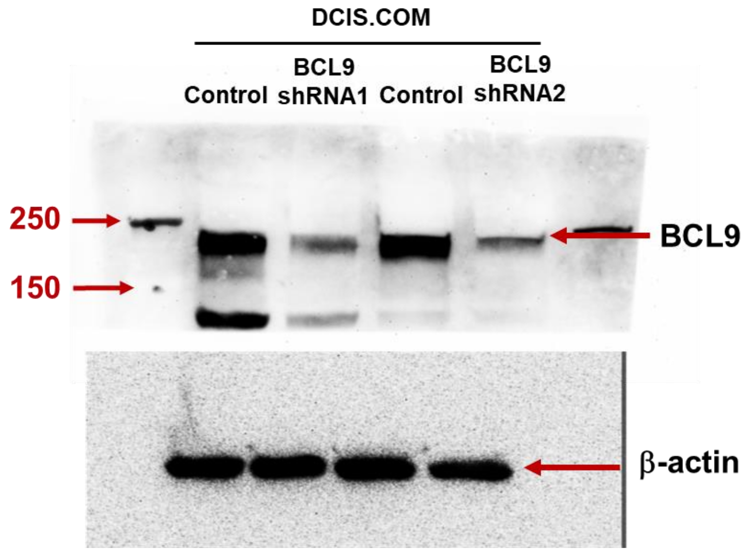

Supplementary Figure 4a

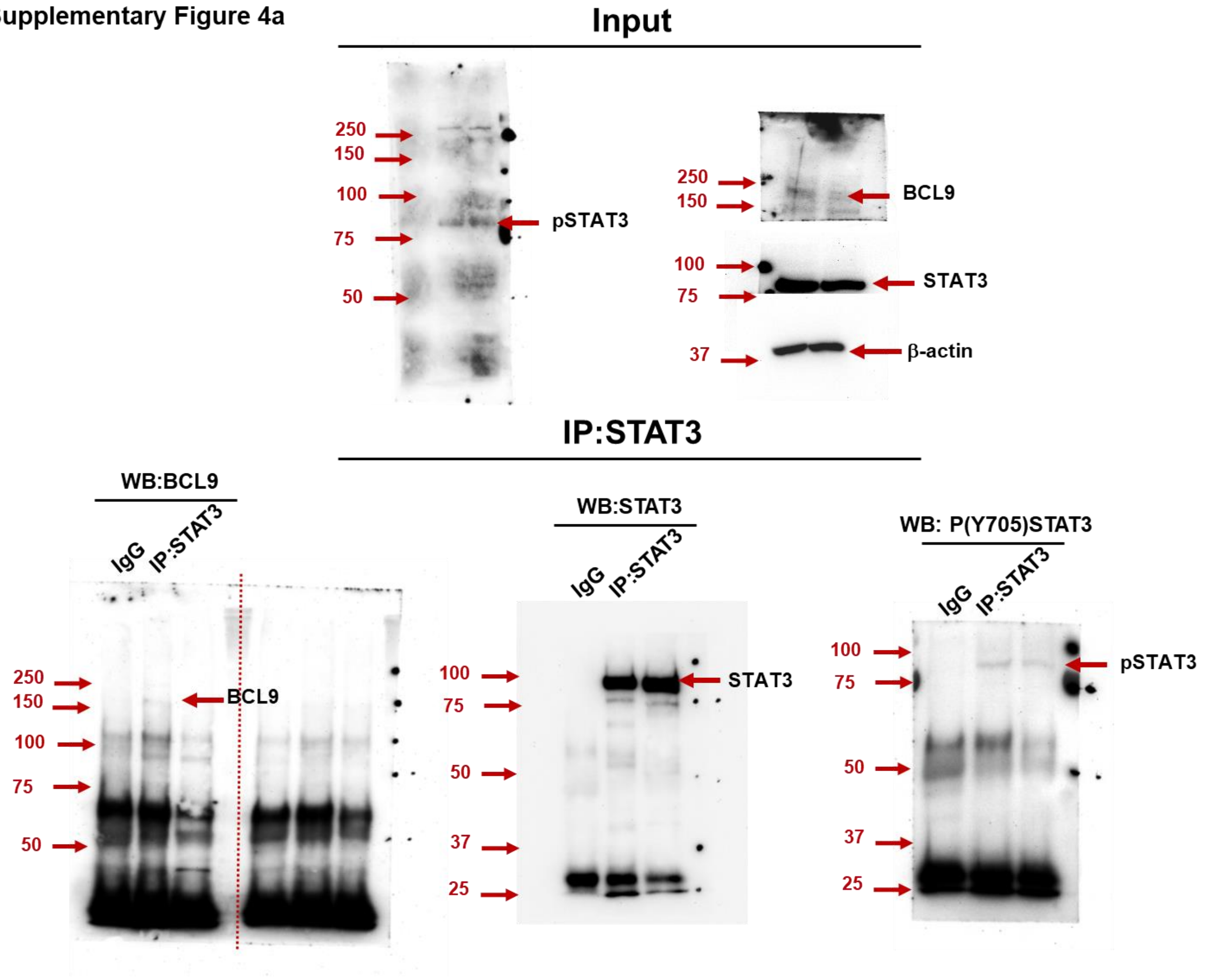

**Supplementary Figure 7c**

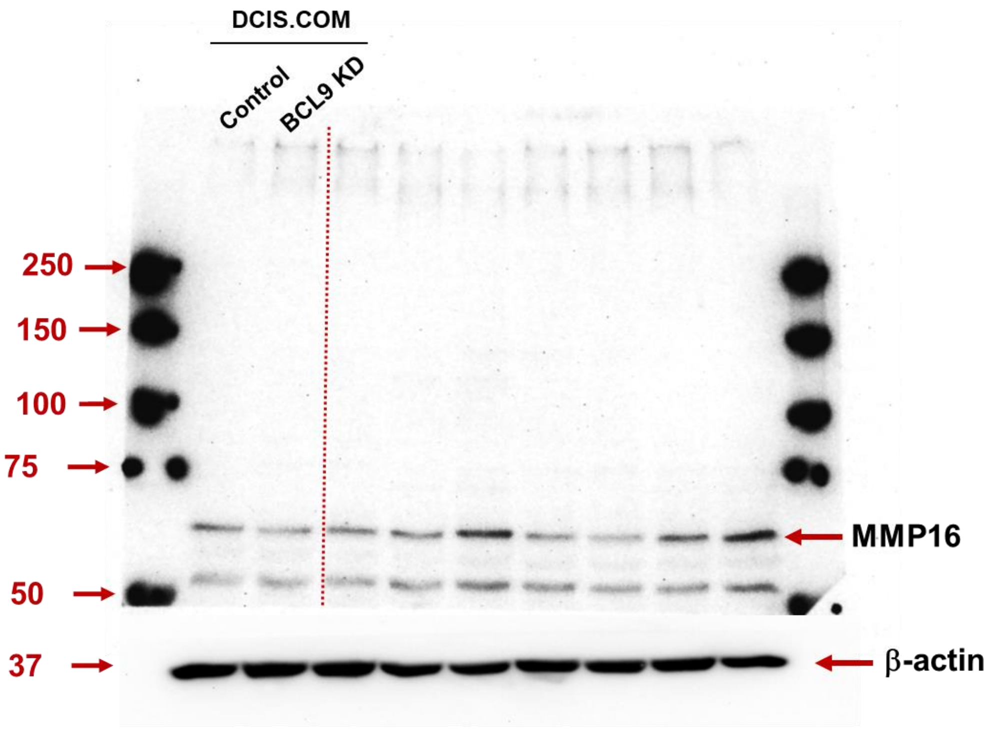


**Supplementary Figure 7d**

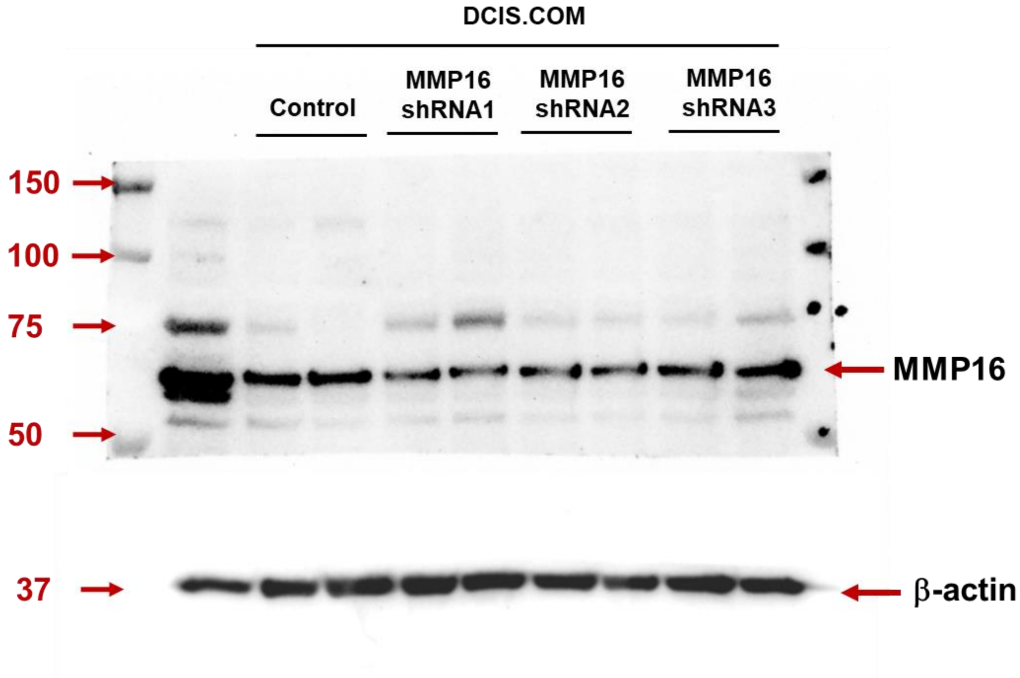

**Supplementary Tables:**

**Supplementary Table 1: List of patient samples included in the progressed versus non-progressed patient-derived MIND xenografts study**

| | Pathology | Grade | ER | PR | HER2 | Ki67 | loss of SMA |
|---|---|---|---|---|---|---|---|
| 1 | DCIS with comedo necrosis and apocrine features | High | 0% | 0% | 0 | 8% | Non-progressed |
| 2 | Solid DCIS with comedonecrosis and microcalcifications | High | 0% | 0% | 1 | 21% | Non-progressed |
| 3 | DCIS solid and cribriform with comedo necrosis | High | 99% | 35% | 0 | 6% | Non-progressed |
| 4 | DCIS cribriform, papillary and micropapillary with comedonecrosis | Intermediate | 96% | 5% | 0 | 3% | Non-progressed |
| 5 | DCIS solid and cribriform types | Low-Intermediate | 99% | 100% | 0 | 1% | Non-progressed |
| 6 | DCIS comedo | High | 90% | 90% | 3 | 33% | Progressed |
| 7 | DCIS solid with comedonecrosis and microcalcifications | Intermediate-High | 99% | 99% | 2 | 10% | Progressed |
| 8 | DCIS comedo with microcalcifications | High | 0% | 0% | 1 | 10% | Progressed |
| 9 | DCIS solid and cribiform with comedo necrosis | High | 99% | 95% | 1 | 15% | Progressed |
| 10 | DCIS comedo and solid type | High | 5% | 0% | 2 | 20% | Progressed |
| 11 | DCIS comedo and solid type with microcalcifications | High | 0% | 0% | 2 | 25% | Progressed |
| 12 | DCIS micropapillary type with microcalcifications | Low-Intermediate | 100% | 40% | 1 | 6% | Progressed |

**Supplementary Table 2**

| | Company | Catalog # | Isotype | Dilution |
|---|---|---|---|---|
| **IF staining** | | | | |
| Primary antibodies | | | | |
| BCL9 | Abcam | ab37305 | Rabbit | 1:50 |
| β-catenin | Abcam | 113110 | Rabbit | 1:50 |
| P(S727)STAT3 | Santa Cruz | sc-24554 | Mouse | 1:50 |
| Integrin αvβ3 | Millipore | MAB1976 | Mouse | 1:50 |
| MMP16 | Abcam | ab73877 | Rabbit | 1:50 |
| Cytokeratin 5 | Vector | VPC400 | Mouse | 1:25 |
| Cytokeratin 19 | Thermoscientific | MS198 | Mouse | 1:50 |
| SMA | Thermoscientific | PA5-18292 | Goat | 1:50 |
| Secondary antibodies | | | | |
| Alexa Fluor 488 anti-rabbit | Invitrogen | A11008 | Goat | 1:200 |
| Alexa Fluor 594 anti-mouse | Invitrogen | A21203 | Donkey | 1:200 |
| Alexa Fluor 488 anti-mouse | Invitrogen | A11012 | Goat | 1:200 |
| **Proximity ligation assay** | | | | |
| BCL9 | Sigma | HPA020274 | Rabbit | 1:50 |
| STAT3 | Cell signaling | 9139S | Mouse | 1:50 |
| P(S727)STAT3 | Santa Cruz | sc-24554 | Mouse | 1:50 |
| β-catenin | BD | 610153 | Mouse | 1:50 |
| Src | Cell signaling | 2109 | Rabbit | 1:50 |
| **Co-immunoprecipitation and ChIP** | | | | |
| IgG | Cell signaling | 2729S | Rabbit | 2 ug |
| BCL9 | Santa Cruz | sc-68915 | Rabbit | 2 ug |
| STAT3 | Santa Cruz | sc-483 | Rabbit | 2 ug |
| Integrin αvβ3 | Millipore | MAB1976 | Mouse | 2 ug |
| Integrin β3 | Santa Cruz | sc-46655 | Mouse | 2 ug |
| **Western blot analysis** | | | | |
| BCL9 | Abcam | ab37305 | Rabbit | 1:1,000 |
| β-catenin | BD | 610153 | Mouse | 1:2,000 |
| β-actin | Chemicon | MAB1501 | Mouse | 1:10,000 |
| P(Y705)STAT3 | Abcam | 113110 | Rabbit | 1:1,000 |
| MMP16 | Abcam | ab73877 | Rabbit | 1:500 |
| FAK | Abcam | ab40794 | Rabbit | 1:500 |
| P(Y397)FAK | Cell signaling | 8556 | Rabbit | 1:1,000 |
| Src | Cell signaling | 2109 | Rabbit | 1:1,000 |
| P(Y416)Src | Cell signaling | 6943 | Rabbit | 1:1,000 |
| Integrin αvβ3 | Millipore | MAB1976 | Mouse | 1:1,000 |
| Integrin β3 | Santa Cruz | sc-46655 | Mouse | 1:1,000 |

**Supplementary Table 3**

| | Sequence/ Assay ID | Company | Catalog # |
|---|---|---|---|
| **ChIP Primers** | | | |
| Downstream 3kb | F GGGGAGTCTGGCTATGACTTTAG<br>R ATGATAACCTCAGATCCCAAGTAGG | IDT | |
| Downstream 10kb | F GAACATAGGCTTTATGAATGACTGG<br>R TGATTCAATGAGTTGTTGTTCTGAG | IDT | |
| Downstream 25kb | F AGTATGTTGAGGTCACTGCCTTG<br>R CATCAGAGGTCAGGAATATAACAGG | IDT | |
| Downstream 44kb | F TGAATTGTGGCAAACATTTCTC<br>R CACTAGTAATGACCTCACGTAGCC | IDT | |
| Downstream 51kb | F GGGTGACATGCCTCTGTTTTAG<br>R TGGGTTCTTTCCCAAAATACTTAGTC | IDT | |
| Downstream 53kb | F TTCATCACCCAGGTAATAAGCATAG<br>R ACAAAGAAGAGAACACCAGACACTG | IDT | |
| Upstream 20kb | F TGTAATAGTCAAGACAGGAAGTGGTC<br>R AATTCTGTGCCACTAGCCTGAG | IDT | |
| Upstream 30kb | F CATCCTAAACAAACTCCGTTGAC<br>R TTTGTCATCTAGTGGTGGAAGGTAG | IDT | |
| **RT-qPCR** | | | |
| Axin2 | Hs00610344_m1 | Thermo Fisher Scientific | 4331182 |
| MMP16 | Hs00234676_m1 | Thermo Fisher Scientific | 4331182 |
| ITGAV | Hs00233808_m1 | Thermo Fisher Scientific | 4331182 |
| ITGB3 | Hs01001469_m1 | Thermo Fisher Scientific | 4331182 |
| MMP2 | Hs01548727_m1 | Thermo Fisher Scientific | 4331182 |
| MMP7 | Hs01042796_m1 | Thermo Fisher Scientific | 4331182 |
| MMP9 | Hs00234579_m1 | Thermo Fisher Scientific | 4331182 |
| MMP11 | Hs00968295_m1 | Thermo Fisher Scientific | 4331182 |
